# The Fifth Adaptor Protein Complex

**DOI:** 10.1371/journal.pbio.1001170

**Published:** 2011-10-11

**Authors:** Jennifer Hirst, Lael D. Barlow, Gabriel Casey Francisco, Daniela A. Sahlender, Matthew N. J. Seaman, Joel B. Dacks, Margaret S. Robinson

**Affiliations:** 1 University of Cambridge, Cambridge Institute for Medical Research, Cambridge, United Kingdom; 2 Department of Cell Biology, University of Alberta, Edmonton, Canada; The Scripps Research Institute, United States of America

## Abstract

Adaptor protein (AP) complexes sort cargo into vesicles for transport from one membrane compartment of the cell to another. Four distinct AP complexes have been identified, which are present in most eukaryotes. We report the existence of a fifth AP complex, AP-5. Tagged AP-5 localises to a late endosomal compartment in HeLa cells. AP-5 does not associate with clathrin and is insensitive to brefeldin A. Knocking down AP-5 subunits interferes with the trafficking of the cation-independent mannose 6-phosphate receptor and causes the cell to form swollen endosomal structures with emanating tubules. AP-5 subunits can be found in all five eukaryotic supergroups, but they have been co-ordinately lost in many organisms. Concatenated phylogenetic analysis provides robust resolution, for the first time, into the evolutionary order of emergence of the adaptor subunit families, showing AP-3 as the basal complex, followed by AP-5, AP-4, and AP-1 and AP-2. Thus, AP-5 is an evolutionarily ancient complex, which is involved in endosomal sorting, and which has links with hereditary spastic paraplegia.

## Author Summary

Adaptor protein (AP) complexes facilitate the trafficking of cargo from one membrane compartment of the cell to another by recruiting other proteins to particular types of vesicles. For over 10 years, it has been assumed that there are four, and only four, distinct AP complexes in eukaryotic cells. We report the existence of a fifth AP complex, AP-5. Immunolocalisation and RNAi knockdown experiments both indicate that AP-5 is involved in trafficking proteins from endosomes towards other membranous compartments. There are genetic links between AP-5 and hereditary spastic paraplegia, a group of human genetic disorders characterised by progressive spasticity in the lower limbs. Phylogenetic analyses indicate that AP-5 was already present in the last eukaryotic common ancestor over a billion years ago.

## Introduction

**Figure 1:**
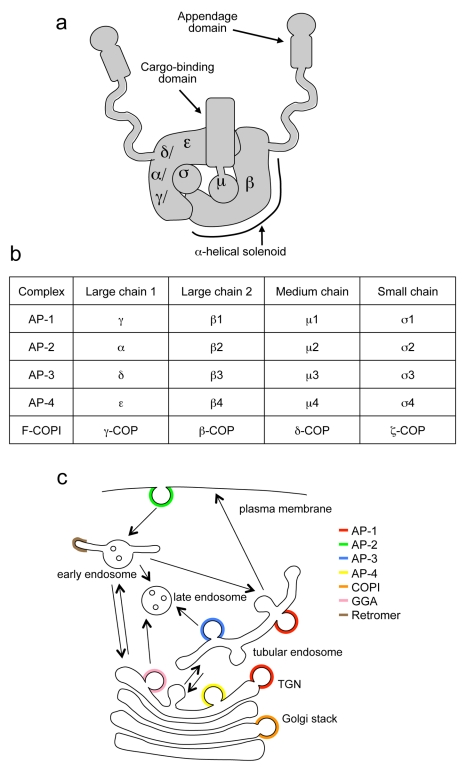
Overview of AP complexes. (a) Structure of an AP complex, showing the positions of the four subunits, and indicating some of the domains on the μ and β subunits. (b) List of subunits in the four AP complexes and F-COPI. (c) Diagram of trafficking pathways and machinery. We have called the AP-1- and AP-3-positive endosomes “tubular endosomes” because this is how they appear by electron microscopy [43],[57]; functional names such as recycling or early endosomes are more contentious.

For many years, it has been assumed that there are four, and only four, adaptor protein (AP) complexes. The first two AP complexes to be identified, AP-1 and AP-2, sort cargo proteins into clathrin-coated vesicles (CCVs). Both AP-1 and AP-2 are heterotetramers, consisting of two large subunits, sometimes called adaptins (γ and β1 in AP-1; α and β2 in AP-2); a medium-sized subunit (μ1 or μ2); and a small subunit (σ1 or σ2) (Figure 1a and b). The AP-3 and AP-4 complexes were discovered by searching databases for homologues of the AP-1 and AP-2 subunits. AP-3 and AP-4 are also heterotetramers, made up of δ, β3, μ3, and σ3 subunits, and of ε, β4, μ4, and σ4 subunits, respectively. However, unlike AP-1 and AP-2, they appear to be able to work without clathrin (reviewed in [1],[2]).

Each of the AP complexes has a distinct localisation and function. AP-1 is localised to tubular endosomes and/or the trans-Golgi network (TGN) and is involved in trafficking between the two organelles, although there is still some uncertainty about the direction [3]. AP-2, the most thoroughly characterised of the four complexes, facilitates clathrin-mediated endocytosis [4]. AP-3 traffics cargo from tubular endosomes to late endosomes, lysosomes, and related organelles, while AP-4 has recently been shown to traffic the amyloid precursor protein from the TGN to endosomes [5]. Thus, all of the AP complexes are involved in post-Golgi trafficking pathways (Figure 1c). In animals, gene knockouts of AP-1 or AP-2 subunits are embryonic lethal [6]. However, animals can survive without AP-3 or AP-4, and mutations in the two complexes in humans have been shown to cause Hermansky Pudlak syndrome [7] and neurological disorders [8]–[10], respectively.

The degree of identity between the related sets of subunits in the four AP complexes is generally in the range of 20%–40%. Another more distantly related heterotetrameric complex is the F subcomplex of the COPI coat (F-COPI), which acts in an earlier pathway, packaging cargo into vesicles for retrograde trafficking from the Golgi apparatus to the ER [1]. F-COPI consists of the large subunits γ-COP and β-COP, the medium subunit δ-COP, and the small subunit ζ-COP. There is also ancient homology between all of the large subunits and between the small subunits and the N-terminal domains of the medium subunits [11]. Although in these cases the sequence identities are no more than ∼10%, the relationship is detectable by sensitive homology searching algorithms, and structural studies show that the proteins adopt very similar folds [12],[13].

This relationship has led to the hypothesis that both complexes evolved from an ancestral heterodimer, consisting of a large chain (the ancestor of all of the large subunits) and a small chain (the ancestor of both medium and small subunits) [1],[11]. This hypothesis is supported by the finding that there are strong interactions between the γ/α/δ/ε-adaptin/γ-COP large subunits and the σ1-4-adaptin/ζ-COP small subunits and between the β1-4-adaptin/β-COP large subunits and the N-terminal domains of the μ1-4-adaptin/δ-COP medium subunits [14],[15]. A further round of gene duplication would have given rise to the F-COPI complex and the proto-AP complex [1],[6].

All of these events must have occurred over one billion years ago, because subunits of all four of the AP complexes, as well as COPI, can be found in genome sequences now available for organisms from across the diversity of eukaryotes. Both homology searching and phylogenetic analyses of sequences strongly suggest that all five complexes were already present in the last common ancestor of all modern eukaryotes [16],[17]. Until now, homology searches of the various databases have not revealed any obvious new family members, suggesting that there were no additional AP complexes to be found.

There are, however, several examples of gene rearrangements to make new proteins with adaptin homology domains. For instance, the GGA proteins, which are monomeric adaptors for clathrin-mediated intracellular trafficking, have a C-terminal domain related to the appendage domain of the γ subunit of AP-1. Three other families of proteins contain μ homology domains (MHDs), which are related to the C-terminal cargo-binding domains of the AP μ subunits. The best characterised of the MHD proteins are the monomeric stonins, which are found only in animals. A second family of MHD proteins includes the mammalian proteins FCHO1, FCHO2, and SGIP1, and the yeast protein Syp1 [1].

The third type of MHD protein is encoded in humans by a gene on chromosome 14 and has been called C14orf108, FLJ10813, or MUDENG (for mu-2-related death-inducing gene, because overexpression of this protein has been reported to cause cell death) [18]. There is unpublished evidence that expression of this gene is upregulated in HIV-1-infected cells (F. Li, submitted to EMBL/GenBank/DDBJ databases, 1998), but otherwise nothing is known about its function. Our interest in the protein initially arose from the observation that a homologue of C14orf108 is present in *Naegleria*, a member of the supergroup Excavata, which diverged from other eukaryotic lineages over a billion years ago [19]. Because so far all MHD proteins have been shown to be involved in membrane traffic, it seemed likely that the same would be true for C14orf108, but with a more ancient function than the GGAs or stonins, which are restricted to opisthokonts. In the present study, we have characterised C14orf108, focussing on its potential binding partners, its localisation, and its function.

## Results

### Domain Organisation of C14orf108

The protein encoded by the C14orf108 gene is 490 amino acids long with a predicted molecular weight of 54.7 kD, similar in size to the μ-adaptins and δ-COP, which range from ∼420–530 residues. Initial BLAST alignments indicated that only the C-terminal MHD of C14orf108 was homologous to the μ-adaptins. However, by performing iterative PSI-BLAST searches, as well as searches using HHpred (http://toolkit.tuebingen.mpg.de/hhpred) [20], we were able to extend the homology further upstream into the N-terminal domains of μ1–4, including the portions that bind to the β subunits.

**Figure 2:**
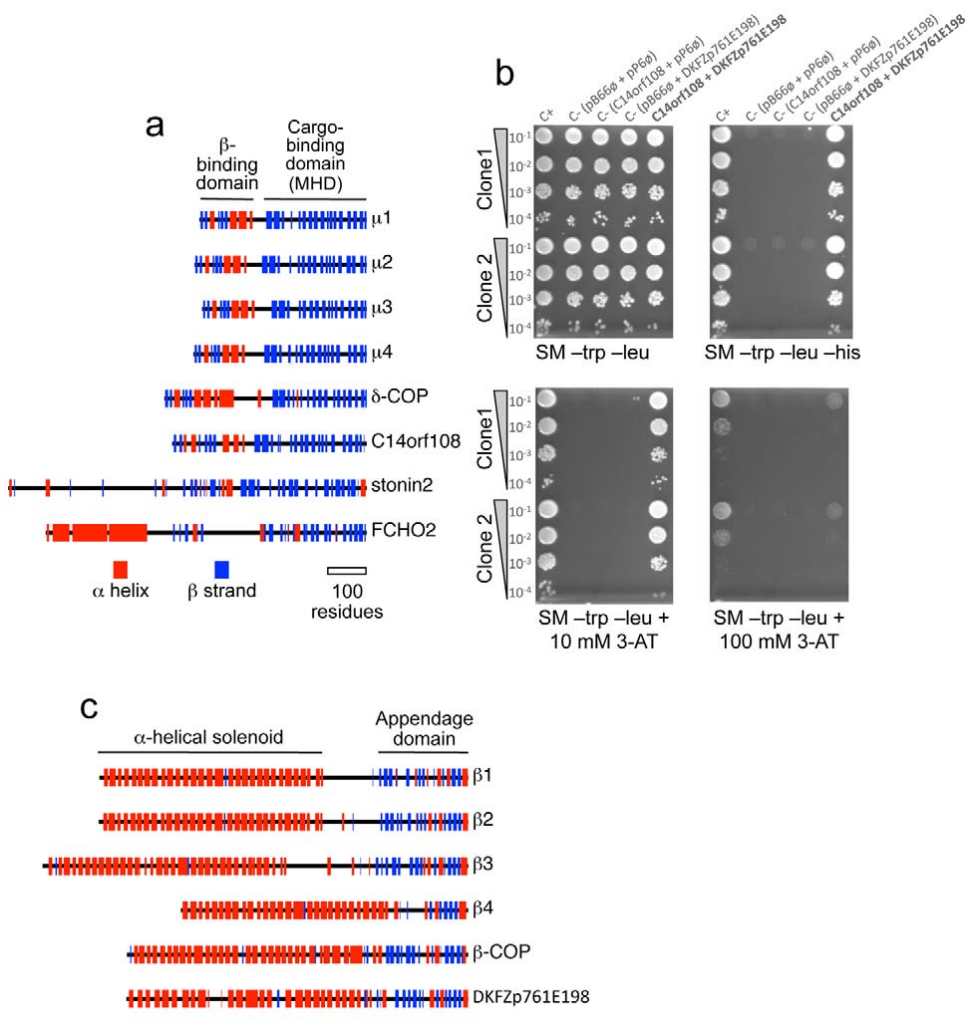
C14orf108 interacts with DKFZp761E198. (a) Secondary structure predictions for the four AP μ subunits, the COPI subunit δ-COP, C14orf108, stonin2, and FCHO2. α helices are shown in red and β strands in blue. The domains in μ1 that bind to the β subunit and to cargo proteins are indicated; similar domains are found not only in the other μ-adaptins and δ-COP, but also in C14orf108. In contrast, stonin2 and FCHO2 share only the cargo-binding (μ-homology) domain (MHD). (b) Yeast two-hybrid interactions between C14orf108 and DKFZp761E198. Two clones encoding residues 16–229 of DKFZp761E198, isolated from a human placental cDNA library screen using C14orf108 as bait, were tested for specificity. “C+” rows contain a positive control; “C−” rows contain various negative controls using the empty bait vector (pB66ø), the empty prey vector (pP6ø), or both. Growth in the absence of histidine (SM –trp –leu –his) or in the presence of 3-aminotriazole (3-AT) indicates that the two gene products interact. (c) Secondary structure predictions for the four AP β subunits, the COPI subunit β-COP, and DKFZp761E198. The α-helical solenoid and appendage domains in β1 are indicated. Similar domains are found in the other family members, and also in DKFZp761E198. The diagram also shows that although DKFZp761E198 lacks a long unstructured linker separating the solenoid and appendage domains, its appendage domain contains both the β-sandwich subdomain and the α/β platform domain, unlike β4 which has only the platform subdomain (or γ, which has only the sandwich subdomain—see Figure 8a).

We also carried out structural predictions using Jpred (http://www.compbio.dundee.ac.uk/www-jpred). The μ-adaptins all have N-terminal domains with a similar fold to the σ-adaptins and to the longin domains of some of the SNARE proteins, consisting of both β strands and α helices. This domain is followed by a short unstructured linker and then the C-terminal cargo-binding domain, a banana-shaped structure composed exclusively of β strands [12],[21]. Figure 2a shows that the predicted secondary structure of C14orf108 is similar to that of the μ-adaptins and δ-COP not only in its C-terminal MHD, but also in its N-terminal domain. In contrast, the two other families of MHD-containing proteins that have been identified, represented in the diagram by stonin2 and FCHO2, have completely different N-terminal domains. These observations suggested to us that C14orf108 might be the μ subunit of a new, previously unsuspected AP complex.

### C14orf108 Binds to a Novel β-Adaptin-Related Protein

To look for binding partners for C14orf108, we used the full-length sequence to screen a human placental yeast two-hybrid library. Out of 69.8 million colonies screened, 136 colonies were isolated. Most of these contained plasmids encoding known proteins with a wide range of functions, and the physiological relevance of their interaction with C14orf108 is unclear. However, there was one uncharacterised sequence derived from a gene encoding a protein called DKFZp761E198, which appeared six times in the screen, although probably as replicates of the same colony, because all of the plasmids encoded only residues 16–229 of the full-length protein. The specificity of the interaction between C14orf108 and DKFZp761E198 was confirmed by repeating the yeast two-hybrid experiment with control sequences inserted into both the bait and the prey vectors (Figure 2b). When the sequence of DKFZp761E198 was analysed using an iterative PSI-BLAST search, the top hits (apart from DKFZp761E198 homologues in other organisms) were all β-adaptins, and HHpred searches also pulled out β-adaptin as the top hit (Table 1). This indicates that like the μ-adaptins and δ-COP, C14orf108 interacts with a β-adaptin-related protein.

**Table 1:** HHpred hits and probability scores.

Protein	Rank	Hit	Prob	Common name
**C14orf108**				
	1	1w63_M	100.0	μ1A (mouse)
	2	2vgl_M	100.0	μ2 (rat)
	3	1i31_A	100.0	μ2 (rat)
**DKFZp761E198**				
	1	2vgl_B	98.4	β2 (rat)
	2	1w63_A	98.2	γ (mouse)
	3	2vgl_A	97.1	α (mouse)
**KIAA0415**				
	1	1w63_A	98.9	γ (mouse)
	2	2vgl_A	98.8	α (mouse)
	3	2vgl_B	98.4	β2 (rat)
**C20orf29**				
	1	2vgl_S	96.8	σ2 (mouse)
	2	2hf6_A	95.9	ζ-COP (human)
	3	1w63_Q	95.8	σ1 (mouse)
**SPG11**				
	1	1xi4_A	85.1	clathrin heavy chain (bovine)
	2	3mkq_A	83.1	β′-COP (yeast)
	3	1xi4_A	70.6	clathrin heavy chain (bovine)

HHpred (http://toolkit.tuebingen.mpg.de/hhpred) is a tool for sequence database searching and structure prediction that looks for proteins of known structure that are homologous to the protein of interest. The table shows the top three HHpred hits for the four AP-5 subunits, as well as the associated protein SPG11. “Prob” refers to the probability for a homologous relationship. The probability is >95% for the four AP-5 subunits and corresponding subunits of AP-1, AP-2, and F-COPI complexes, and >80% for SPG11 and two structural coat proteins, clathrin heavy chain and β′-COP. The same clathrin heavy chain hit (1xi4_A) appears twice for SPG11 because of alignments of different regions of SPG11 with different regions of clathrin heavy chain (residues 1644–2268 with residues 337–766; and residues 1200–1806 with residues 567–1362).

The corrected full-length sequence of human DKFZp761E198 is 878 amino acids long, corresponding to a predicted protein size of 94 kD (the sequence in the database is missing the N-terminal 57 residues). This is in line with the β-adaptin/β-COP subunits, which range in size from ∼740–1100 residues. In addition, Jpred secondary structure predictions reveal that DKFZp761E198 is structurally very similar to a β-adaptin or β-COP. Like all of the adaptor large subunits, the β-adaptins consist of a long N-terminal α-helical solenoid, followed by an unstructured flexible linker and a C-terminal appendage domain, usually comprising a β-sandwich subdomain followed by a mixed α-β subdomain. Figure 2c shows that DKFZp761E198 adopts a very similar fold, although the flexible linker is much shorter. The mapping of the binding site on DKFZp761E198 for C14orf108 to residues 16–229 is also consistent with a β-μ-like interaction, because the structures of both AP-2 and AP-1 reveal that the N-terminal quarter of the β subunit makes most of the contacts with the μ subunit [12],[13].

Although C14orf108 and DKFZp761E198 are clearly homologous to the AP μ and β subunits, respectively, analysis of their sequences indicates that they differ in several ways from most other μ and β family members. The key residues in the μ subunits that bind to YXXΦ sorting signals [21] are altered in C14orf108, suggesting that if C14orf108 is involved in cargo recognition, it probably interacts with a different type of motif. In addition, the classical clathrin binding box found in the flexible linker region of some of the β-adaptins [22] is missing in DKFZp761E198, and it also lacks two other clathrin-binding motifs, LLDLL and YQW. DKFZp761E198 does contain a copy of the fourth clathrin-binding motif, WDW; however, this sequence is in the middle of the α-helical solenoid and is therefore unlikely to interact with clathrin.

### Localisation of C14orf108

**Figure 3:**
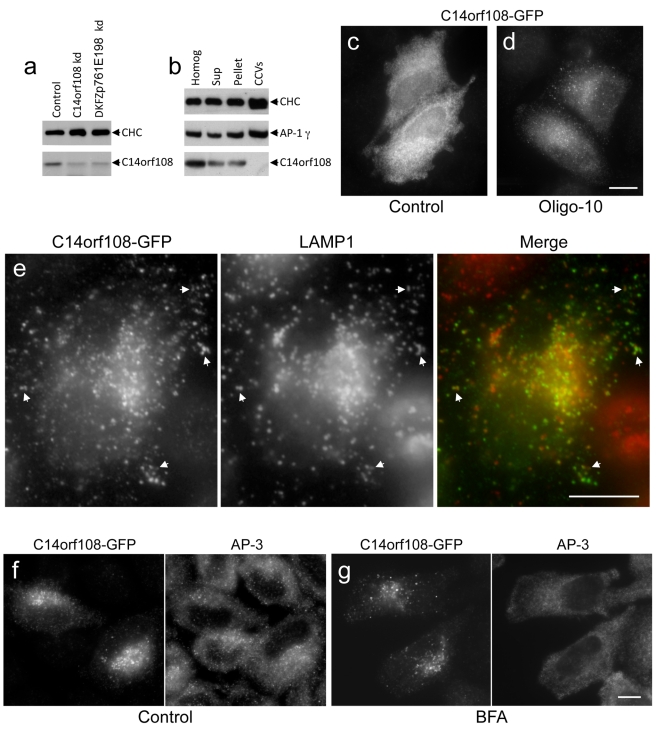
Characterisation of C14orf108 by Western blotting and immunofluorescence. (a) A rabbit antiserum raised against a C14orf108-derived fusion protein recognises a band of the appropriate size on Western blots of HeLa cell homogenates, which is reduced in intensity when the cells are treated with SMARTpool siRNAs targeting the protein, and also when cells are treated with SMARTpool siRNAs targeting DKFZp761E198. For this and all subsequent siRNA experiments, control cells were treated with RISC-free control siRNA. (b) C14orf108 partitions between membranes and cytosol when cell homogenates are centrifuged at high speed, and does not appear to be associated with clathrin-coated vesicles (CCVs). (c) In cells transfected with GFP-tagged C14orf108, most of the construct is cytosolic. (d) If the cells are treated with Oligo-10, an siRNA that depletes endogenous C14orf108, the tagged construct has a punctate distribution in a limited number of cells. (e) Double labelling for tagged C14orf108 and LAMP1 in cells treated with Oligo-9. By moderating protein levels, treatment with Oligo-9 highlights the membrane association of the construct. There is substantial overlap between tagged C14orf108 and LAMP1. (f and g) Cells treated with Oligo-9 were double labelled for tagged C14orf108 and AP-3, either without (f) or with (g) a 5-min incubation in 20 µM brefeldin A (BFA). Unlike AP-3, C14orf108 is insensitive to BFA. The need to use anti-GFP, instead of relying on GFP fluorescence, indicates that AP-5 is not very abundant. Scale bars: 20 µm.

To investigate the subcellular distribution of C14orf108, we first raised antisera against recombinant constructs. The μ-adaptins are generally very poor immunogens, but we have succeeded in raising antibodies against the linker regions of μ1 and μ2 that work for Western blotting, although not for immunofluorescence [14]. Thus, we made a fusion protein incorporating residues 165–205 of C14orf108 and used it to immunise rabbits. One of the antisera recognised a band of the appropriate size (∼55 kD) on Western blots (Figure 3a). The identity of this band was confirmed when we treated the cells with siRNAs targeting C14orf108 and found that the intensity of the signal was greatly reduced. Interestingly, knocking down DKFZp761E198 also decreased the intensity of the C14orf108 band, indicating that, like AP μ and β subunits [23], C14orf108 is stabilised by binding to DKFZp761E198.

Although our antibody did not work for immunofluorescence, we were able to gain some insights into the localisation of C14orf108 by cell fractionation followed by Western blotting. Blots of whole HeLa cell homogenates, high-speed supernatants, membrane-containing pellets, and a CCV-enriched fraction were probed with antibodies against C14orf108, clathrin, and the AP-1 γ subunit (Figure 3b). Like clathrin and AP-1, C14orf108 partitions approximately equally between membranes and cytosol, indicating that it cycles on and off the membrane; but unlike clathrin and AP-1, it is not enriched—or even detectable—in the CCV fraction.

As an alternative to immunofluorescence localisation of endogenous C14orf108, we made several tagged constructs, adding a GFP tag to the N or C terminus, or inserting a myc tag between the N- and C-terminal domains. However, when these constructs were transfected into HeLa cells, the transfection efficiency was poor, expression levels were low, and only cytoplasmic labelling was seen, even after saponin extraction (Figure 3c). Attempts to make stably transfected cell lines were unsuccessful, possibly because exogenously expressed C14orf108 may be toxic [18]. We were also unable to raise specific antibodies against DKFZp761E198 (but see below) or to localise epitope-tagged DKFZp761E198.

The exclusively cytosolic labelling that we saw with tagged C14orf108 is inconsistent with our Western blotting results, which indicate that at least some of the endogenous C14orf108 is associated with membranes. We speculated that the exogenous tagged constructs might be competing unsuccessfully with the endogenous protein for membrane docking sites and/or for coassembly with DKFZp761E198 to make a functional complex. Therefore, we depleted cells of endogenous C14orf108 using single siRNA oligonucleotides and then transfected in GFP-tagged C14orf108. One of the siRNAs that we used, Oligo-10, targets the 3′UTR and therefore only depletes the endogenous C14orf108. Under these conditions, we still saw mainly cytosolic labelling; however, in some of the cells we could also detect a punctate pattern (Figure 3d).

A second siRNA, Oligo-9, targets both endogenous and tagged proteins, and as expected, expression of our construct was reduced in cells treated with Oligo-9 (Figure S1). Strikingly, however, in all of the GFP-positive cells (visualised using anti-GFP), we could now see punctuate labelling that was concentrated in the perinuclear region of the cell (Figure 3e–g). To try to identify the C14orf108-positive compartment, we double labelled with markers for different types of endosomes and for the TGN, as well as with antibodies against clathrin and other coat components. Although in most cases we saw little or no overlap (Figure S1), there was considerable overlap with LAMP1, a marker for late endosomes and lysosomes (Figures 3e and S1; see below for quantification). This colocalisation with LAMP1 suggests that C14orf108 may function in the late endocytic pathway.

Most of the Golgi and post-Golgi coat components, including COPI, AP-1, AP-3, AP-4, and GGAs, are acutely sensitive to the drug brefeldin A (BFA). However, when we treated cells expressing GFP-tagged C14orf108 with BFA, we saw no detectable change in the localisation pattern, even though double labelling for other coats (e.g., AP-3, which also associates with an endosomal compartment) showed redistribution into the cytosol (Figure 3f and g). The insensitivity of C14orf108 to BFA suggests that its recruitment onto membranes does not depend on ARF GTPases, or at least not on ARFs that are recruited onto membranes by a BFA-sensitive guanine nucleotide exchange factor.

### RNAi Knockdown Phenotype

**Figure 4:**
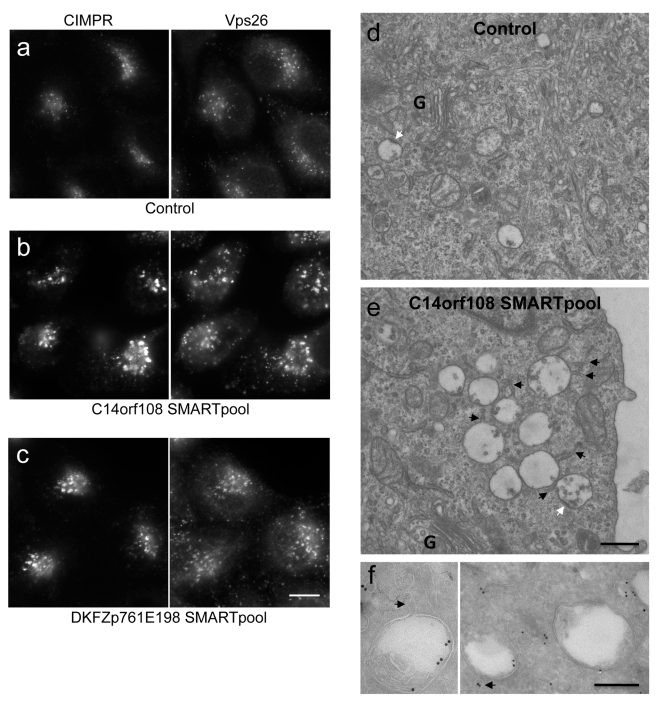
Phenotype of siRNA-treated cells. (a–c) Double labelling for the CIMPR and the retromer subunit Vps26 in control cells (a), cells depleted of C14orf108 (b), and cells depleted of DKFZp761E198 (c). Both knockdowns cause a change in the localisation of the CIMPR and Vps26: the individual puncta are larger and brighter, and there are fewer of them per cell (see Figure 8c for quantification). Scale bar: 20 µm. (d and e) Electron micrographs of the Golgi (G) region of control (d) and C14orf108-depleted (e) cells. Knocking down C14orf108 causes the cells to accumulate swollen multivesicular bodies (MVBs), which have tubules emanating from them (black arrows) and flat clathrin bilayer coats (white arrows). Scale bar: 500 nm. (f) Immunogold labelling of C14orf108-depleted cells. The MVBs are positive for the CIMPR, indirectly labelled with 15 nm (left) or 10 nm (right) gold particles. The arrows indicate tubules and vesicles emanating from the MVBs. Scale bar: 200 nm.

To learn more about C14orf108 function, we depleted the endogenous protein using siRNA, and then looked for defects in endocytic trafficking. We saw no changes in the uptake of transferrin from the cell surface, or in the distribution of LAMP1 (unpublished observations). However, there was a striking change in the localisation of the cation-independent mannose 6-phosphate receptor (CIMPR), a receptor for lysosomal hydrolases that cycles between the TGN and endosomes. In control cells, the CIMPR localises to fine puncta that show some overlap with the retromer protein Vps26 (a marker for early endosomes) (Figure 4a). When C14orf108 is depleted, both the CIMPR and Vps26 localise to much larger puncta in the perinuclear region of the cell (Figures 4b and S2). This phenotype was observed with individual siRNAs as well as with the “SMARTpool” (containing four individual siRNAs), indicating that it is not an off-target effect (Figure S2).

We also treated the cells with SMARTpool and individual siRNAs targeting DKFZp761E198. Again, the CIMPR redistributed to larger puncta in the perinuclear region of the cell, where it colocalised with Vps26 (Figure 4c and Figure S3; see below for quantification). The finding that the DKFZp761E198 knockdown phenocopies the C14orf108 knockdown provides further evidence not only that the knockdown phenotypes are specific, but also that the interaction between C14orf108 and DKFZp761E198 is physiologically relevant.

To look for changes in the siRNA-treated cells at the ultrastructural level, we carried out both conventional and immunogold electron microscopy. In conventional thin sections, we saw an increase in multivesicular bodies (MVBs) near the Golgi apparatus (Figure 4d and 4e). These MVBs appeared to be swollen and often had tubules emanating from them (black arrows). The presence of flat clathrin bilayer patches on the MVBs (white arrows) indicates that they are a relatively early endosomal compartment. Immunogold labelling for the CIMPR showed that gold particles were associated with these structures, sometimes with the limiting membrane but more often with neighbouring or emanating tubules and vesicles (Figure 4f). Together with the C14orf108 localisation data, these observations point to a role for the C14orf108-DKFZp761E198 complex in endosomal trafficking.

### Phylogenetic Distribution and Evolution of C14orf108 and DKFZp761E198

In order to investigate whether the C14orf108-DKFZp761E198 complex is unique to humans and related species, or whether it is a more general feature of eukaryotic cells, we performed comparative genomics using sensitive homology searching methodology. Twenty-nine eukaryotic genomes were searched, spanning the five major supergroups that encompass eukaryotic diversity (treating the contentious SAR/CCTH groups [24] as a single unit). Candidate sequences were classified as homologues of subunits of one of the four known AP complexes or of the novel complex, based initially on a positive homology searching result using the reciprocal best hit criteria (see Materials and Methods for details). Several additional candidate DKFZp761E198 homologues were identified as being reciprocally retrieved in PSI-BLAST searches (Table S1).

After preliminary analyses to identify highly divergent and/or near-identical sequences (Figures S4 and S5), phylogenetic analyses were performed on the μ-adaptin homologues and on the β-adaptin homologues. The μ homologues were robustly resolved into the known AP complexes, with an additional clade of C14orf108 homologues strongly supported (Figure S6). The analyses of β homologues provided modest support for a clade of DKFZp761E198 homologues (Figure S7). Finally, a concatenated phylogenetic analysis of the β- and μ-adaptin subunits (Figure S8) showed very high support values for clades encompassing each of the four known AP complexes and a fifth clade (1.0/89%/72%) containing all of the putative C14orf108 and DKFZp761E198 homologues. Therefore, we suggest that C14orf108 and DKFZp761E198 should be renamed μ5 and β5, respectively, and that the complex that they form should be called AP-5.

**Figure 5:**
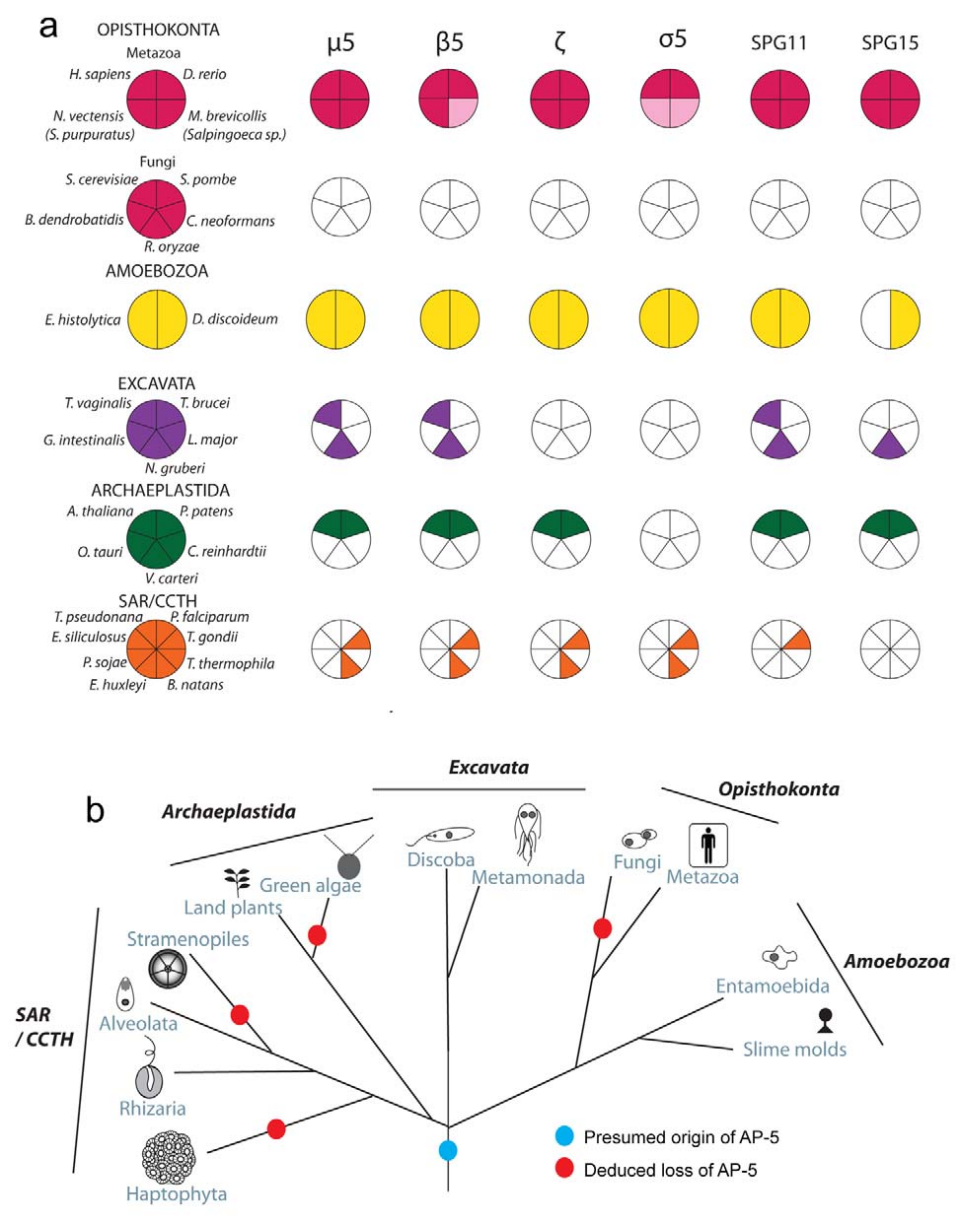
Distribution and proposed evolution of the AP-5 complex in eukaryotes. (a) Coulsen plot with the coloured sectors denoting the presence of μ5, β5, ζ, σ5, SPG11, and SPG15 homologues in 12 of the 29 genomes sampled, spanning the extent of eukaryotic diversity. The light pink shaded sections in Metazoa indicate that an orthologue was not identified in *N. vectensis* or *M. brevicollis* but was identified in the nr database from the taxon listed parenthetically. (b) Deduced evolutionary history of the AP-5 complex as present in the Last Eukaryotic Common Ancestor but lost multiple times independently.

Overall, candidate homologues were identified for μ5 and β5 in at least two representatives from each of the 5 major eukaryotic supergroups (Figure 5a). This suggests that the AP-5 complex is found as a broadly conserved feature of eukaryotic cells, but has been lost on multiple occasions. It also suggests that AP-5 was an ancient feature of eukaryotic cells (Figure 5b).

**Figure 6:**
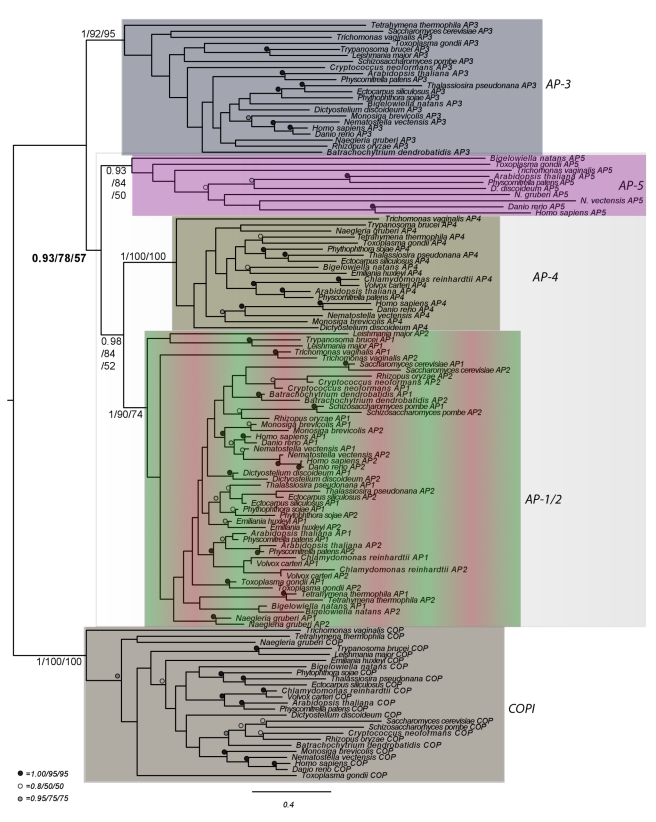
Order of evolutionary emergence for the adaptin protein families. Phylogenetic analysis of a concatenated dataset of β-adaptin and μ-adaptin homologues from diverse eukaryotes, rooted with the β and δ COP homologues. The different complexes are colour coded in the same way as in Figures 1c and 10. AP-3 is demonstrated here to be the earliest diverging adaptor complex, highlighted by the shaded box showing its exclusion from the other AP families. The best Bayesian topology is shown with support values listed in the order of Posterior probability values and ML bootstrap support values for PhyML and RAxML. Numerical values are given for the backbone nodes and for the monophyly of each protein family (illustrated by the shaded boxes). Other values are replaced with symbols as inset.

**Figure 7:**
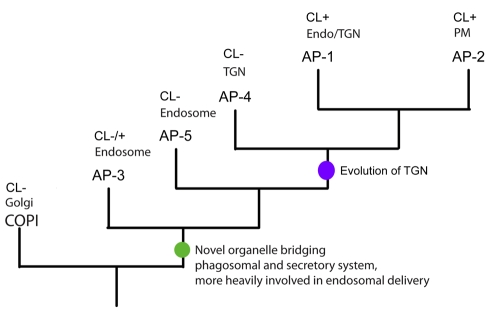
Schematic drawing illustrating the order of duplications giving rise to the adaptor complexes. The green circle denotes the hypothetical origin of a primordial hybrid organelle linking the secretory system and the endocytic system, while the purple circle denotes the specialisation into a primordial TGN compartment.

Most excitingly, analysis of the concatenated β and μ dataset, with the COP sequences used as an outgroup, provided the first robust evolutionary order of emergence for the adaptin complexes (Figure 6). Taking into account previous data showing that AP-1 and AP-2 were the most recent complexes to evolve [17], we can now hypothesize that AP-3 is the basal adaptor complex, followed by AP-5, AP-4, and then finally AP-1 and AP-2 (Figure 7).

### Search for Other Subunits

The four AP complexes and the F-COPI complex are all heterotetramers, suggesting that AP-5 might also be a heterotetramer, containing not only μ5 and β5, but also another large subunit and a small subunit. In the case of AP-1 and AP-2, the β subunit interacts with the other large subunit in the yeast two-hybrid system [14], so we carried out a yeast two-hybrid library screen using β5 as bait, but did not find any candidates for another adaptin. However, interactions between the two large subunits of an AP complex are not always detectable in the yeast two-hybrid system: we have previously tried and failed to show yeast two-hybrid interactions between β3 and δ [23] and between β4 and ε [25]. We also carried out iterative PSI-BLAST searches and HMMer searches to try to find the potential missing subunits, but again found no promising hits (unpublished data).

Co-immunoprecipitation would be another way to find additional subunits, but unfortunately our antibody against endogenous μ5/C14orf108 was unable to immunoprecipitate, and so far we have not succeeded in generating enough cells expressing GFP-tagged μ5 for biochemical experiments. However, Słabicki et al. [26] recently identified both DKFZp761E198/β5 and C14orf108/μ5, together with another uncharacterised protein, C20orf29, in immunoprecipitations that they performed using tagged versions of three different proteins: KIAA0415, SPG11, and SPG15. KIAA0415 was found in an RNAi library screen for genes involved in DNA repair, and SPG11 and SPG15 are two proteins that are mutated in hereditary spastic paraplegia (HSP). Although the significance of the DNA repair phenotype is unclear, several of the HSP genes encode proteins known or thought to be involved in membrane traffic [27]. In addition, Słabicki et al. provide evidence that KIAA0415 is also mutated in some patients with HSP and suggest that the protein should be called SPG48.

**Figure 8:**
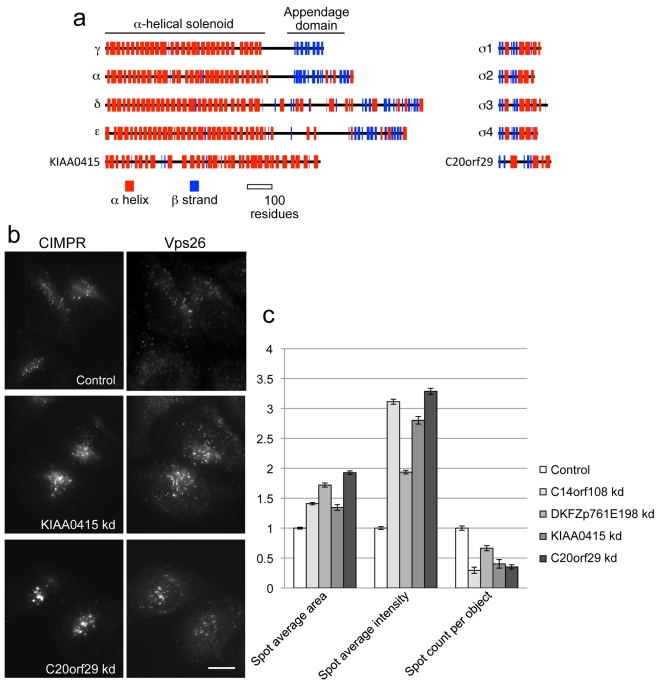
Candidates for other subunits of the AP-5 complex. (a) KIAA0415/SPG48 and C20orf29, which have been shown to coimmunoprecipitate with μ5 and β5 [26], have similar predicted secondary structures to the γ/α/δ/ε large subunits and to the small subunits, respectively, although KIAA0415/SPG48 lacks an appendage domain and C20orf29 has longer loops between the folded domains. (b) Knockdown of either KIAA0415/SPG48 or C20orf29 phenocopies μ5 and β5 knockdowns, causing both the CIMPR and Vps26 to localise to larger puncta. Scale bar: 20 µm. (c) Quantification of the changes in CIMPR immunofluorescence observed in cells treated with siRNAs targeting each of the four putative subunits. The changes were quantified using an automated microscope and normalised to the control. In every case, the spots are larger and brighter, and there are fewer of them per cell.

**Figure 9:**
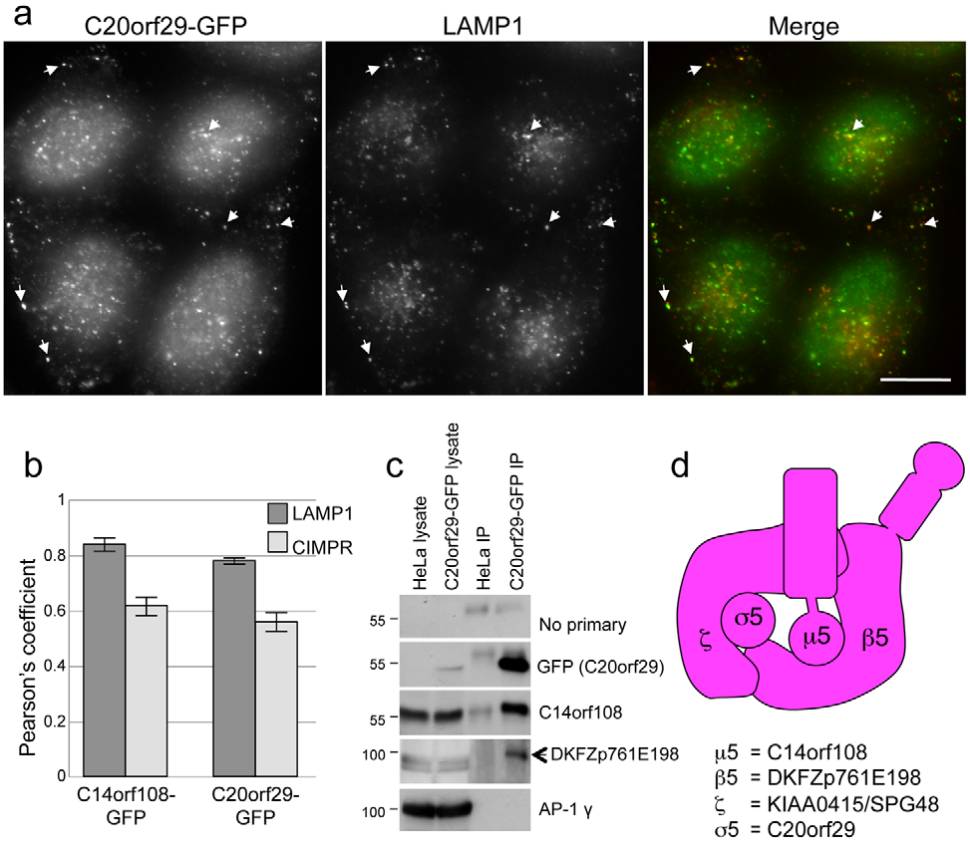
Further evidence for a heterotetrameric complex. (a) Localisation of GFP-tagged C20orf29. Transiently transfected cells were fixed, extracted with saponin, and double labelled for GFP and LAMP1. There is substantial overlap between the two proteins. (b) Quantification of overlap. The level of overlap between either C14orf108-GFP or C20orf29-GFP, and either LAMP1 and the CIMPR, was quantified using Volocity software, from which Pearson's correlation coefficient was determined. The Pearson's coefficients were 0.850±0.023 for C14orf108-GFP and LAMP1; 0.627±0.029 for C14orf108-GFP and the CIMPR; 0.789±0.003 for C20orf29-GFP and LAMP1; and 0.565±.032 for C20orf29-GFP and the CIMPR. (c) Immunoprecipitation of AP-5 subunits from cells expressing GFP-tagged C20orf29. Control HeLa cells and cells expressing C20orf29-GFP were lysed and immunoprecipitated with anti-GFP, then probed with antibodies against various proteins. In the cells expressing the construct, the anti-GFP antibody not only brings down the construct itself, but also C14orf108 and DKFZp761E198. The anti-DKFZp761E198 antibody produces a high background of non-specific bands that are unaffected by siRNA knockdown, but it labels a band of the expected size (arrow) after the complex is enriched by immunoprecipitation. The blot was also probed with an antibody against the AP-1 γ subunit as a control to ensure that the immunoprecipitation was specific. (d) Proposed organisation of the AP-5 complex, conforming to the established nomenclature for AP subunits: we are calling the other large subunit (KIAA0415/SPG48) ζ, the next letter in the Greek alphabet after ε; and we are calling the small subunit (C20orf29) σ5.

Interestingly, several features of KIAA0415/SPG48 and C20orf29 suggest that they may be the other large subunit and the small subunit of the AP-5 complex. First, they are the right size: KIAA0415/SPG48 is 807 amino acids long, and C20orf29 is 200 amino acids long. Second, KIAA0415/SPG48 has a similar predicted secondary structure to the γ/α/δ/ε subunits, although it lacks a C-terminal appendage domain, and C20orf29 has a similar predicted secondary structure to the σ subunits, but with longer loops connecting the folded domains (Figure 8a). Third, iterative PSI-BLAST and HHpred searches using KIAA0415/SPG48 and C20orf29 identify AP large subunits and small subunits, respectively, as principal homologues (Table 1). Fourth, knocking down either KIAA0415/SPG48 or C20orf29 produces the same phenotype as knocking down μ5 or β5: both the CIMPR and Vps26 accumulate in a “chunky” perinuclear compartment (Figures 8b, S9, and S10), and quantification of the labelling shows that knocking down any of the four proteins increases the size and relative intensity of the spots, and decreases the number of spots per cell (Figure 8c). Fifth, homology searches identified homologues of both KIAA0415/SPG48 and C20orf29 in nearly all of the taxa found to possess C14orf108/μ5 and DKFZp761E198/β5 (see Figure 5a), although phylogenetic analysis was required to confirm the affinity of one C20orf29 homologue (Figure S11). Sixth, we have been able to localise GFP-tagged C20orf29 without needing to use siRNA to “damp down” expression levels, although saponin extraction was necessary to reduce the cytosolic background, and we see the same pattern as with GFP-tagged C14orf108. In both cases, there was relatively little colocalisation with most markers, but substantial colocalisation with LAMP1 (Figure 9a and b). Finally, because a much higher percentage of cells expressed GFP-tagged C20orf29 than GFP-tagged C14orf108, we were able to immunoprecipitate the construct with anti-GFP and then probe Western blots with antibodies against the other subunits. Figure 9c shows that anti-C14orf108/μ5 labels a band of the expected size, and moreover that an antibody that we generated against DKFZp761E198/β5, which we thought was unusable because it labelled multiple non-specific bands on Western blots of whole cell homogenates, also labels a band of the expected size on our Western blot of the immunoprecipitate, indicating that it is capable of working on β5-enriched samples. These results provide independent confirmation that the three proteins that were detected in immunoprecipitates with antibodies against tagged KIAA0415 [26] also come down with antibodies against another tagged subunit.

Thus, we propose that like the other AP complexes, AP-5 is a heterotetramer, consisting of two large subunits, a medium subunit, and a small subunit (Figure 9d). We also propose that like other AP complexes [28], AP-5 binds accessory proteins, two of which are SPG11 and SPG15.

## Discussion

The current paradigm in intracellular trafficking is that there are four AP complexes, each mediating a different post-Golgi pathway. Additional homology has been reported between the AP complexes and the COPI F subcomplex, and there are also several proteins with adaptin homology domains, including C14orf108 [1]. However, until now, the relationship between C14orf108 and the μ-adaptins has been unclear.

Here we show that C14orf108 has more in common with the μ-adaptins than with other MHD-containing proteins such as the stonins and FCHO1/2. C14orf108's homology to the μ-adaptins extends upstream beyond the MHD, and secondary structure predictions indicate that the μ-adaptins and C14orf108 adopt very similar folds. In addition, C14orf108 binds to a previously uncharacterised protein, DKFZp761E198, which is homologous to the β-adaptins and has an almost identical predicted secondary structure. Further evidence for the interaction between C14orf108 and DKFZp761E198 comes from the destabilisation of C14orf108 when DKFZp761E198 is knocked down, from their similar knockdown phenotypes, and from the taxonomic distribution of the two genes. In eukaryotes from five different supergroups, the genes are always found either together or not at all, suggesting coordinate loss. For all of these reasons, we have proposed that C14orf108 and DKFZp761E198 are the μ5 and β5 subunits of a novel complex, AP-5. While this work was in progress, Słabicki et al. [26] demonstrated that μ5 and β5 can be coimmunoprecipitated with two other novel proteins, KIAA0415/SPG48 and C20orf29. These two proteins have a number of properties that suggest that they are the other large subunit (ζ) and the small subunit (σ5) of the AP-5 complex, including similar knockdown phenotypes to μ5 and β5.

How can we reconcile the results of the present study with the results of Słabicki et al., who identified KIAA0415 in a screen for DNA repair genes? The DNA repair phenotype appears to be robust and on-target, because the authors were able to rescue it with RNAi-resistant KIAA0415. However, the phenotype could be indirect. For instance, Słabicki et al. also pulled out a Rho GEF, ARHGEF1, in their screen, and they proposed that it might have a signalling role which, when disrupted, could affect DNA repair, e.g. by interfering with protein phosphorylation. A similar scenario could apply to KIAA0415, since there are many connections between cell signalling and membrane traffic, especially in the endocytic pathway [29]. The authors also proposed that KIAA0415 might be a helicase, based on a bioinformatics analysis. However, our own data argue against such a role. We have localised tagged versions of two of the subunits to endosomes; knockdowns affect endosomal trafficking; and HHpred searches with KIAA0415 pull out AP large subunits as the top hits (Table 1). Further down the list of hits are other proteins with α-helical solenoids, such as importins, but there are no helicases on the list. Furthermore, helicases have a number of motifs that are essential for enzymatic activity [30], and these motifs are missing in KIAA0415, indicating that KIAA0415 is unlikely to be a helicase.

In addition to the other candidate AP-5 subunits, Słabicki et al. coimmunoprecipitated SPG15 and SPG11 with KIAA0415. SPG15 and SPG11 are two proteins that are mutated in hereditary spastic paraplegia (HSP), and both proteins have features that are consistent with a role in the AP-5 pathway. First, the taxonomic distributions of these two proteins closely match those of the four proposed AP-5 subunits (Figure 5a). Second, SPG15 has a FYVE domain, a zinc finger domain that binds to the endosomal phosphoinositide phosphatidylinositol 3-phosphate (PI3P), and like other FYVE domain-containing proteins, SPG15 has been localised to endosomes [31],[32]. Similarly, many of the proteins associated with endocytic CCVs are recruited onto the membrane by binding to the plasma membrane phosphoinositide, phosphatidylinositol 4,5-bisphosphate (PIP2), so SPG15 may be another component of the AP-5-containing coat, such as an “alternative adaptor” [33]. Third, when SPG11 was analysed by HHpred, the top hit was clathrin heavy chain, followed by β′-COP, a component of the COPI coat which, like clathrin, is thought to drive vesicle formation by assembling into a cage-like structure (Table 1) [34]. Thus, SPG11 may be a component of the outer part of the AP-5-containing coat, possibly acting as a membrane-deforming scaffold.

**Figure 10:**
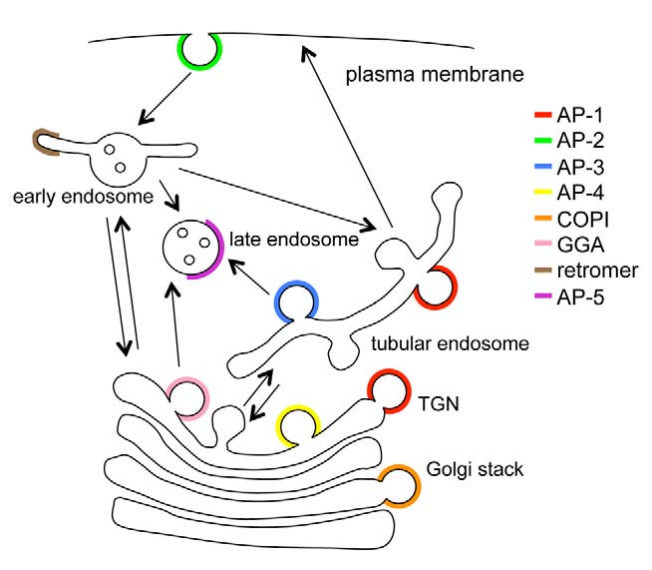
Updated diagram of trafficking machinery and pathways. AP-5 is unusual in that it is the only coat identified so far that associates with late endosomes.

What is the function of AP-5? Western blotting indicates that it cycles on and off membranes but is not associated with CCVs. The lack of any identifiable clathrin binding sites in β5 and the lack of colocalisation between tagged AP-5 and clathrin also indicate that AP-5 is clathrin-independent. In addition, the lack of key residues in μ5 for binding YXXΦ motifs indicates that, if AP-5 is an adaptor, it must be recognising some other type of sorting signal, in the same way that the MHD protein stonin recognises a non-canonical sorting signal on the membrane protein synaptotagmin [1]. The partial colocalisation of tagged μ5 with LAMP1, the altered appearance of the CIMPR and Vps26 in AP-5-depleted cells, and the accumulation of swollen MVBs in such cells all point to a role in the endocytic pathway. However, the compartment that is morphologically altered in AP-5-depleted cells is positive for the CIMPR and Vps26, and AP-5 does not show much colocalisation with either of these proteins. Together, these observations suggest that the site of action of AP-5 is the late endosome and/or lysosome, as defined by the presence of LAMP1 (Figure 10), but that when it is depleted, there are indirect effects on earlier, retromer-positive endosomes, possibly because a bottleneck is created. Although trafficking out of late endosomes has never been formally established, there are a number of late endosomal membrane proteins that need to be recycled, such as receptors for lysosomal hydrolases and different types of SNAREs [35], so AP-5 may be part of a coat that facilitates vesicle budding from this compartment. So far, relatively little machinery has been identified for the later stages of the endocytic pathway. A protein complex associated with the yeast vacuole was recently discovered, called the SEA complex, which also has some features of a coat, although structurally it is more closely related to tethering complexes [36]. Thus, at present the AP-5 complex appears to be the best candidate for a late endosomal coat. The connection with hereditary spastic paraplegia, a group of genetic disorders that already have a number of links with membrane traffic [27], provides a promising lead for future investigations into AP-5 function.

Based on the taxonomic distribution of AP-5, we deduce that it is an ancestral complex, but that it has been lost frequently throughout eukaryotic history. In most instances we observed coordinated absence of all six proteins examined. However, in a few instances we failed to identify one or more subunits in specific taxa. These missing subunits could indicate that the complex is in the process of being lost in those lineages. Investigation of publicly available databases, however, showed that in *Arabidopsis*, *Physcomitrella*, and *Toxoplasma*, nearly all of the identified AP-5 subunits were expressed (unpublished data), arguing instead for bioinformatic false negatives in the homology searches of the missing components. Indeed, sensitive homology searching was needed in many cases to identify the AP-5 homologues, which are clearly divergent sequences in phylogenetic analysis (Figures 6, S4–S8, and S11). In the case of SPG11 and SPG15, alternate accessory proteins might be in use in diverse eukaryotic lineages, as recently described for the endosomal trafficking complex retromer [37],[38].

Nonetheless, AP-5 represents a most extreme case of the more general evolutionary expendability of adaptor complexes, as AP-2, AP-3, and AP-4 have each been lost from various organisms [1],[39]–[42]. This may reflect the evolutionary plasticity of the endocytic machinery, which needs to be adapted to diverse life strategies, and it could potentially reflect the functional overlap seen between the adaptor complexes (e.g., see [43]). The toxic nature of μ5 when overexpressed in mammalian cells [18] could provide some hints for the basis of the repeated loss of the complex.

We have previously suggested that the duplication giving rise to COPI and the ancestral adaptor complex was coincident with the origin of the Golgi proper and the TGN [17]. Given the involvement of the various adaptor complexes with the endocytic system, particularly the basally emerging AP-3 and AP-5 complexes, we now speculate that this duplication may well have correlated with the specialisation of a Golgi compartment (COPI-associated) and a primordial endosome/TGN compartment, representing a first integration of the phagosomal endocytic pathway and the secretory pathway (Figure 7). Hypothetically, this primordial endosome/TGN with both secretory and endocytic features would later further expand to become the various endosomes and the TGN (Figure 7).

Why wasn't the AP-5 complex discovered earlier? One reason is that AP-5 does not appear to be present in several of the major model organisms used to study membrane traffic, such as *Saccharomyces cerevisiae*. In addition, AP-5's resemblance to the rest of the family is relatively weak. Even when comparing the same AP-5 subunit in closely related species like humans and mice, the degree of conservation is surprisingly low. For instance, human and mouse β2 are 99.9% identical: there is only a single conservative amino acid substitution in the 951-residue protein. In contrast, human and mouse β5 are only 85% identical: when one aligns the two sequences, there are 136 amino acid substitutions and four gaps. More generally, as seen from the phylogenetic analyses (Figures 6, S4–S8, and S11), the AP-5 components always represent divergent sequences, pointing to a lack of selective pressure on AP-5, which has made the subunits very difficult to identify even with sophisticated bioinformatic techniques. But although over 10 years have elapsed since the last AP complex, AP-4, was discovered [25],[44], it now appears that what we all thought was the “final recount” of adaptins [1],[33] was not so final after all and that there may be additional surprises in store.

## Materials and Methods

### Internet Tools

Consensus secondary structure predictions and sequence alignments were performed using the Multialign and ClustalW programs (http://npsa-pbil.ibcp.fr/) and Jpred (http://www.compbio.dundee.ac.uk/www-jpred/index.html). Molecular visualisation was performed using RasMol (http://www.umass.edu/microbio/rasmol/). Protein family (Pfam) domains are listed in the Pfam Homepage (http://pfam.wustl.edu/) and in the PROSITE Homepage (http://www.expasy.org/prosite/). Functional sites were predicted using the Eukaryotic Linear Motif (ELM) resource (http://elm.eu.org/), and molecular weights predicted using EXPASy Compute pI Tool (http://expasy.org/tools/pi_tool.html). Homology searches were carried out using PSI-BLAST (http://blast.ncbi.nlm.nih.gov/Blast.cgi) and HHpred (http://toolkit.tuebingen.mpg.de/hhpred).

### Antibodies

Antisera against C14orf108 were raised in rabbits against a GST fusion of amino acids 165–205. This sequence was amplified from EST IMAGE clone 7775556 and cloned in-frame into pGEX4T-1. Antisera against DKFZp761E198 were raised in rabbits against a GST fusion of amino acids 661–878. This sequence was amplified from a full-length ORIGENE clone RC214265 and cloned in-frame into pGEX4T-1. Mouse monoclonal antibodies against CIMPR and LAMP1 were purchased from AbCam and Santa Cruz, respectively. Other antibodies used in this study were raised in-house and have been described elsewhere [45],[46]. Secondary antibodies for immunofluorescence were purchased from Invitrogen.

### Yeast Two-Hybrid Interactions

A commercial yeast two-hybrid library screen was carried out by Hybrigenics (Paris, France). The coding sequence for the full-length human C14orf108 protein (GenBank accession number gi: 21361775) was PCR-amplified and cloned in frame with the LexA DNA-binding domain (DBD) into plasmid pB27 (orientation N-LexA-C14orf108-C) and subcloned into pB66 as a C-terminal fusion to Gal4 DNA-binding domain (N-Gal4-C14orf108-C). pB27 and pB66 derive from the original pBTM116 [47] and pAS2ΔΔ [48] plasmids, respectively. The DBD constructs were checked by sequencing the entire inserts.

A prey fragment corresponding to a previously undescribed transcript variant of the human DKFZp761E198 protein was extracted from the ULTImate Y2H screening of full-length C14orf108 against a human placenta cDNA library. This fragment was cloned in frame with the Gal4 activation domain (AD) into plasmid pP6, derived from the original pGADGH [49]. The AD construct was checked by sequencing the insert at its 5′ and 3′ ends.

The “one-by-one” yeast two-hybrid interaction assay used here is based on the reporter gene HIS3. The DBD constructs were transformed into L40DGal4 yeast cells and the AD constructs into Y187 yeast strain. The following interaction pairs were then tested using a mating approach as previously described [48]: i) Hybrigenics' positive control; ii) Empty pB66 – empty pP6 (negative control); iii) C14orf108 – empty pP6 (negative control); iv) Empty pB66 – DKFZp761E198 (negative control); v) C14orf108 – DKFZp761E198.

Interaction pairs were tested in duplicate as two independent clones from each mating reaction were picked for the growth assay. For each interaction, several dilutions (10^−1^, 10^−2^, 10^−3^, and 10^−4^) of the diploid yeast cell culture normalized at 5×10^4^ cells and expressing both bait (DBD fusion) and prey (AD fusion) constructs were spotted on several selective media. The selective medium lacking tryptophan and leucine (SM –trp-leu) was used as a growth control. The different dilutions were also spotted on a selective medium without tryptophan, leucine, and histidine (SM –trp-leu-his). Different concentrations of 3-aminotriazole (3-AT), an inhibitor of the HIS3 gene product, were added to the selective plates to increase stringency and reduce possible autoactivation by the bait proteins. For simplicity only the 10 mM and 100 mM concentrations of 3-AT tested are shown.

### RNA Interference

To deplete C14orf108 and DKFZp761E198, we obtained ON-TARGET plus SMARTpool siRNAs (Dharmacon), and used the pools combined at 100 nM or the deconvoluted single oligos at 25 nM. For controls we used siGENOME RISC-free control siRNA (Dharmacon).

For C14orf108 (NM_018229) the siRNAs are J-015523-09 ORF (Oligo-9), J-015523-10 3′-UTR (Oligo-10), J-015523-11 ORF (Oligo-11), and J-015523-12 ORF (Oligo-12).

For DKFZp761E198 the siRNAs are J-015530-09-005 3′-UTR (Oligo-9), J-015530-09-005 ORF (Oligo-10), J-015530-09-005 3′-UTR (Oligo-11), and J-015530-09 ORF (Oligo-12).

For KIAA0415 and c20orf29, we used the ON-TARGET plus SMARTpool reagents LU-025284-01 and LU-021239-02 (Dharmacon), respectively. We also used the deconvoluted single oligos J-025284-17, J-025284-18, J-025284-19, and J-025284-20 for KIAA0415; and J-021239-17, 021239-18, 021239-19, and 021239-20 for C20orf29.

### Tissue Culture, Immunolocalization, Western Blotting, and Immunoprecipitations

HeLa M cells [50] were maintained in DMEM supplemented with 10% fetal calf serum, 2 mM L-glutamine, 50 units/ml penicillin, and 50 µg/ml streptomycin at 37°C. For transfection with tagged AP-5, PCR was used to amplify the coding sequence of C14orf108 or C20orf29, removing the stop codon and adding restriction sites so it could be ligated into pEGFPN vectors, and the cells were then transiently transfected and viewed after 48 h. For siRNA knockdowns, cells were seeded at 25% confluency and transfected with siRNA 3 h later using Oligofectamine (Invitrogen), as specified by the manufacturer. The transfection mix was left on for 48 h and the cells assayed 72 h after transfection. For immunofluorescence, cells were plated onto glass-bottomed dishes (Mattek) and fixed with 3% paraformaldehyde followed by permeabilisation with 0.1% Triton ×100. The cells were imaged with a Zeiss Axiovert 200 inverted microscope using a Zeiss Plan Achromat 63× oil immersion objective, a Hamamatsu ORCA-ER2 camera, and IMPROVISION OPENLAB software. The level of colocalisation between different fluorescently labelled proteins was quantified using Volocity software.

To quantify knockdown phenotypes, we used an automated ArrayScan VTI microscope (Cellomics/Thermo-Fisher) and the SpotDetector V4 assay algorithm. Cells were plated onto 96-well Perkin Elmer microplates and stained with anti-CIMPR followed by Alexa Fluor 488-donkey anti-mouse IgG and whole cell stain (Invitrogen). The cells were imaged with a modified Zeiss Axiovert 200 M inverted microscope, a Zeiss 40×/0.5 Achroplan objective, and a Hamamatsu OCRA-ER camera, and >1,500 cells quantified using ARRAYSCAN software.

Isolation of CCVs and probing of Western blots of cell fractions were carried out as previously described [45],[51]. For immunoprecipitations, cells expressing either C14orf108-GFP or C20orf29-GFP were solubilised in PBS containing 1% NP40, insoluble material removed, and the samples were pre-cleared with Protein-A-Sepharose (GE Healthcare). For C14orf108-GFP, the cells were then immunoprecipitated with an antibody against GFP followed by recovery with Protein-A-Sepharose. For C20orf29-GFP, the GFP antibody was directly coupled to the resin using NHS-activated Sepharose 4 Fast Flow (GE Healthcare).

For immunogold labelling, control and siRNA treated HeLa cells were fixed by adding an equal volume of freshly prepared 4% paraformaldehyde/0.4% gluteraldehyde in 0.1 M phosphate buffer, pH 7.4. After 5 min the solution was removed and cells were postfixed in 2% paraformaldehyde/0.2% glutaraldehyde in 0.1 M phosphate buffer, pH 7.4, for 1 h at room temperature and further processed as previously described [52]. Ultrathin sections were labelled with mouse anti-CIMPR antibody followed by a rabbit anti-mouse antibody (Dako) and detected with protein A conjugated to colloidal gold (Utrecht University, Utrecht, the Netherlands). Conventional plastic thin sections of cells were also prepared, as previously described [51]. In both cases, the grids were viewed using a Phillips CM 100 transmission electron microscope (Philips Electron Optics, Cambridge, United Kingdom) at an operating voltage of 80 kV.

### Homology Searching

Databases from organisms spanning the breadth of eukaryotic diversity were downloaded from various websites including the National Center for Biotechnology Information (NCBI) (www.ncbi.nlm.nih.gov), the Joint Genome Institute (JGI) (www.jgi.doe.gov/), the Broad Institute (www.broadinstitute.org), the Sanger Institute (www.sanger.ac.uk), or from the URLs given. The URL details and listing of taxa sampled in this study are found in Table S1. Additional searches of the non-redundant database at NCBI were performed. As detailed in Figure 5a, *Strongylocentrotus purpuratus* was deemed as a proxy for *N. vectensis*, while *Salpingoeca* sp. was deemed as a proxy for *M. brevicollis*, representing basal animals and Choanoflagellata, respectively.

Initial BLASTp searches for homologues of C14orf108, DKFZp761E198, KIAA0415/SPG48, and C20orf29 or of the known μ-adaptin, β-adaptin, δ-COP, and β-COP subunits used the human sequence as the search query into the respective databases. Genomes were also searched using the putative C14orf108, DKFZp761E198, KIAA0415, and C20orf29 homologues from the closest related organism. For further sensitivity, PSI-BLAST searches were also performed using the human sequence as the initial query and including retrieved sequences that met the reciprocal BLAST criteria (see below). Additionally, Hidden Markov Model (HMM) searches were performed using HMMer, with subunit-specific HMMs built containing C14orf108, DKFZp761E198, KIAA0415, and C20orf29 orthologues only or else retrieved sequences for all AP complexes in order to identify any unidentified adaptins in the relevant genome of interest.

In all cases, candidate sequences with E-values 1.0 or better were then reciprocally used as queries for BLASTp or PSI-BLAST searches of the *Homo sapiens* database. Sequences were considered validated if they retrieved the *H. sapiens* homologue with E-values better than 0.05 or a homologue in a related genomic database that had in turn retrieved the human homologue. The exception was homologues from *T. vaginalis* and *E. histolytica*, which was subsequently validated by phylogenetic analysis (Figures S8, S11). All accession numbers for sequences are found in Table S1.

### Phylogenetics

To address the evolution of the AP complexes, several datasets were constructed. An initial dataset of all μ-adaptin homologues included 131 taxa and 364 amino acid positions. After subsequent removal of highly divergent sequences and highly similar taxon-specific homologues, a final μ dataset consisted of 110 taxa and 364 positions. The initial dataset of β-adaptin homologues contained 126 sequences and 550 positions. After removal of divergent sequences, taxon-specific duplicates, and putative DKFZp761E198 that did not meet the reciprocal BLAST criteria, the remaining dataset contained 103 taxa and 550 positions. A concatenation of AP β and μ sequences was also performed. Some organisms have independently duplicated their AP-1 and AP-2 β genes, while others possess a single gene that acts as both β1 and β2 [17]. In the case of the latter, the β1/β2 gene was inputted to the concatenated alignment twice as the homologous sequence to both β1 and β2. An initial dataset of adaptins contained 109 sequences and 906 positions and included all taxa sampled with the exception of *Plasmodium falciparum* and *Giardia intestinalis*, as these taxa consistently presented long branches in the previous analyses. A dataset rooted with the δ- and β-COP sequences, and with *Entamoeba histolytica* sequences removed due to ambiguity in the orthology assignments of adaptins from this taxon, was constructed containing 125 sequences and 906 positions. Finally, a dataset (46 sequences, 135 positions) of σ-adaptin sequences from taxa putatively possessing C20orf29 was investigated to confirm orthology of candidate σ5 homologues. All alignments are available from the authors upon request.

Sequences were aligned using MUSCLE (http://www.drive5.com/muscle/muscle.html) and manually adjusted. Only positions of unambiguous homology were included for phylogenetic analysis. ProtTest V.10.2 [53] was used to select a best-fit model incorporating rate among site and invariant site correction when relevant. Subsequently, Mr. Bayes 3.1.2 [54] was used to produce the optimal topology and posterior probability values. Analyses were run for 1–5×10^6^ Markov Chain Monte Carlo (MCMC) generations and a burn-in value was determined by graphical estimation and removal of all trees appearing before the graphical plateau. In the case of the rooted concatenated analysis (Figure 6), the analysis was run for ∼6.7×10^6^ generations and then restarted using the optimal resulting topology as a user defined starting tree for an additional ∼4.5×10^6^ generations until convergence was achieved. Burnin was assessed in this case using the sump command, and in all cases the trees collected prior to the splits frequency descending below 0.1 were omitted. Additionally, RAxML version 7.0 [55] and PhyML version 2.44 [56] were used to obtain maximum likelihood bootstrap values (100 pseudoreplicates).

## Supporting Information

Figure S1 Conditions for immunofluorescence and localisation of GFP-tagged C14orf108. (a) Immunoprecipitation (using anti-GFP) of extracts from control cells and cells transiently transfected with C14orf108-GFP, either with or without knocking down C14orf108 with Oligo-9, which targets both endogenous and tagged C14orf108. Western blots of the immunoprecipitates and a homogenate of non-transfected cells were probed with anti-C14orf108. The construct is strongly reduced after knockdown, and this allows the finer details of the labelling to be seen. (b–g) Double labelling for GFP-tagged C14orf108 and other proteins in Oligo-9-treated cells. There is little or no colocalisation between tagged C14orf108 and the cation-independent mannose 6-phosphate receptor (CIMPR), Vps26 (a retromer subunit associated with early endosomes), TGN46 (a TGN protein), clathrin heavy chain (CHC), or the AP-1 adaptor complex; however, substantial colocalisation can be seen between tagged C14orf108 and LAMP1, a protein associated with late endosomes and lysosomes. Scale bar: 20 µm.
(TIF)

Figure S2 Phenotype of cells depleted of C14orf108 using either a “SMARTpool” mixture of four siRNAs or each siRNA individually. (a–f) The SMARTpool and Oligos 9, 10, and 12 all cause a similar change in the localisation of both the CIMPR and Vps26. Scale bar: 20 µm. (g) Knockdown efficiency assayed by Western blotting. Oligo-11, the only siRNA that does not change the appearance of the CIMPR, is also the least efficient of all the oligos at depleting C14orf108. Scale bar: 20 µm.
(TIF)

Figure S3 Phenotype of cells depleted of DKFZp761E198 using either a SMARTpool mixture of four siRNAs, or each siRNA individually. All four of the individual siRNAs, as well as the SMARTpool, change the localisation of the CIMPR in a similar manner to the C14orf108 siRNAs. Scale bar: 20 µm.
(TIF)

Figure S4 Phylogenetic analysis of all identified μ-adaptin homologues. In this and all subsequent figures the best Bayesian topology is shown, and values are given in the order of Bayesian posterior probabilities, PhyML derived maximum likelihood bootstrap values, and RAxML derived maximum-likelihood bootstrap values for the backbone nodes and those defining the adaptin protein families. Other values are replaced with symbols as inset.
(TIF)

Figure S5 Phylogenetic analysis of all identified β-adaptin homologues.
(TIF)

Figure S6 Phylogenetic analysis of μ-adaptin homologues with highly divergent and lineage-specific high identity duplicates removed. Note the robust support for the clade of μ5 orthologues.
(TIF)

Figure S7 Phylogenetic analysis of β-adaptin homologues with highly divergent and lineage-specific high identity duplicates removed and including only putative homologues of β5 that were clearly identified using the PSI-BLAST criteria.
(TIF)

Figure S8 Phylogenetic analysis of a concatenated dataset of μ- and β-adaptin homologues. This encompasses all putative AP-5 homologues and robustly shows a clade of AP-5, thus solidifying their orthology.
(TIF)

Figure S9 Phenotype of cells depleted of KIAA0415 using individual siRNAs instead of the SMARTpool. All four of the siRNAs change the localisation of the CIMPR. Scale bar: 20 µm.
(TIF)

Figure S10 Phenotype of cells depleted of C20orf29 using individual siRNAs instead of the SMARTpool. (a) All four of the siRNAs change the localisation of the CIMPR. Scale bar: 20 µm. (b) The same four siRNAs were tested on cells transiently expressing GFP-tagged C20orf29, and the blot was probed with anti-GFP. The two siRNAs that target the coding sequence, Oligo-19 and Oligo-20, also deplete the GFP construct; however, Oligo-17 and Oligo-18, which target the 3′ UTR, do not deplete the construct because it has a different 3′ UTR.
(TIF)

Figure S11 Phylogenetic analysis σ-adaptin homologues. Note the robust grouping of the putative σ5 orthologues including *E. histolytica* AP5S1 (XP_001914013.1), which retrieved the *D. discoideum* σ5 homologue as the most significant hit but with an e-value above the cut-off (0.082), thus validating the *E. histolytica* orthology as a σ5.
(TIF)

Table S1 Information for all sequences used in phylogenetic analysis or positively identified by homology searching. Table lists by **taxon** (with genome project URL provided); our proposed **annotation**; database identifier (Genbank accession number, whenever possible); **name** used in our sequence alignments for ease of comparison between raw datasets, databases, and figures; and **reciprocal** E-value for the BLAST search of the sequence to the relevant human homologue or validated homologue in taxonomic nearest relative as the top scoring retrieved sequence.
(XLS)

## Abbreviations

AP: adaptor protein
BFA: brefeldin A
CCV: clathrin-coated vesicle
CIMPR: cation-independent mannose 6-phosphate receptor
HSP: hereditary spastic paraplegia
MHD: μ-homology domain
MVB: multivesicular body
TGN: trans-Golgi network
