# Correction: The Fifth Adaptor Protein Complex

**DOI:** 10.1371/annotation/89dff893-c156-44bb-a731-bfcc91843583

**Published:** 2012-03-26

**Authors:** Jennifer Hirst, Lael D. Barlow, Gabriel Casey Francisco, Daniela A. Sahlender, Matthew N. J. Seaman, Joel B. Dacks, Margaret S. Robinson

The following information was missing from the Funding section: This study was supported by a grant from Alberta Innovates Technology Futures (AITF).

