# Supplementary figures and images for: The Fifth Adaptor Protein Complex
Source: PLoS Biol. 2011 Oct 11;9(10):e1001170. doi: 10.1371/journal.pbio.1001170 (PMC3191125; doi:10.1371/journal.pbio.1001170)

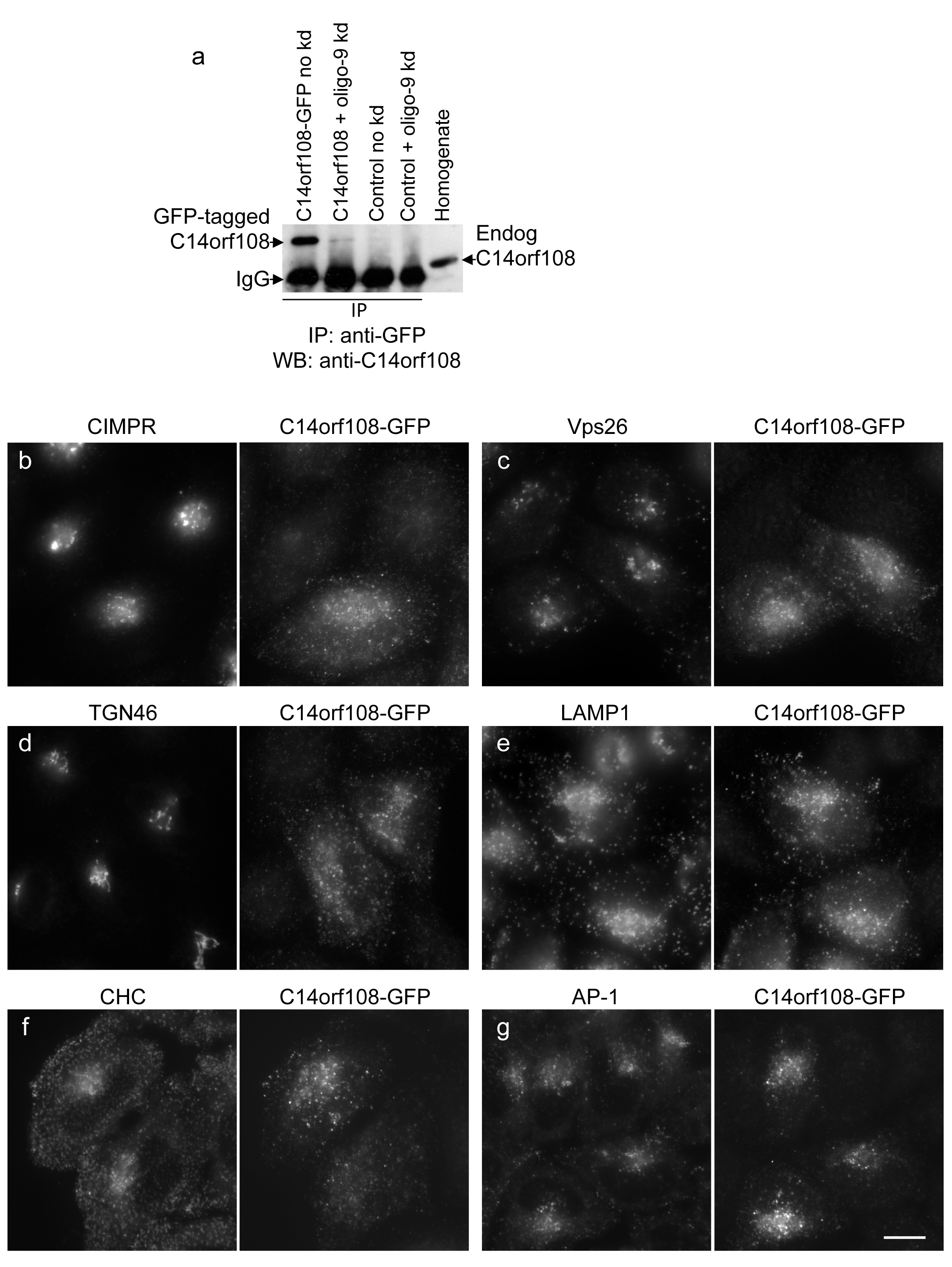

Supplement: Figure S1 — Conditions for immunofluorescence and localisation of GFP-tagged C14orf108. (a) Immunoprecipitation (using anti-GFP) of extracts from control cells and cells transiently transfected with C14orf108-GFP, either with or without knocking down C14orf108 with Oligo-9, which targets both endogenous and tagged C14orf108. Western blots of the immunoprecipitates and a homogenate of non-transfected cells were probed with anti-C14orf108. The construct is strongly reduced after knockdown, and this allows the finer details of the labelling to be seen. (b–g) Double labelling for GFP-tagged C14orf108 and other proteins in Oligo-9-treated cells. There is little or no colocalisation between tagged C14orf108 and the cation-independent mannose 6-phosphate receptor (CIMPR), Vps26 (a retromer subunit associated with early endosomes), TGN46 (a TGN protein), clathrin heavy chain (CHC), or the AP-1 adaptor complex; however, substantial colocalisation can be seen between tagged C14orf108 and LAMP1, a protein associated with late endosomes and lysosomes. Scale bar: 20 µm. (TIF) [file pbio.1001170.s001.tif]

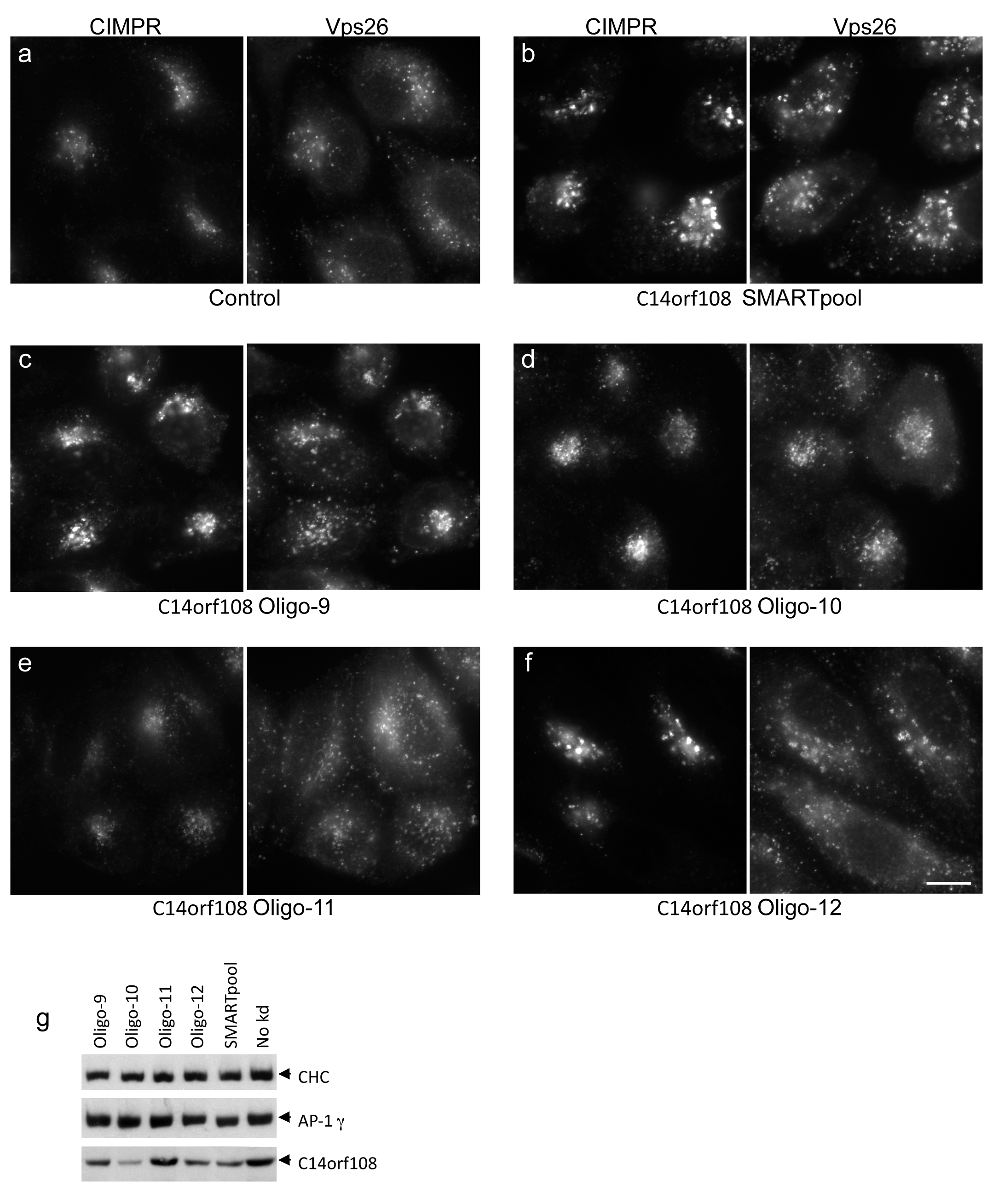

Supplement: Figure S2 — Phenotype of cells depleted of C14orf108 using either a “SMARTpool” mixture of four siRNAs or each siRNA individually. (a–f) The SMARTpool and Oligos 9, 10, and 12 all cause a similar change in the localisation of both the CIMPR and Vps26. Scale bar: 20 µm. (g) Knockdown efficiency assayed by Western blotting. Oligo-11, the only siRNA that does not change the appearance of the CIMPR, is also the least efficient of all the oligos at depleting C14orf108. Scale bar: 20 µm. (TIF) [file pbio.1001170.s002.tif]

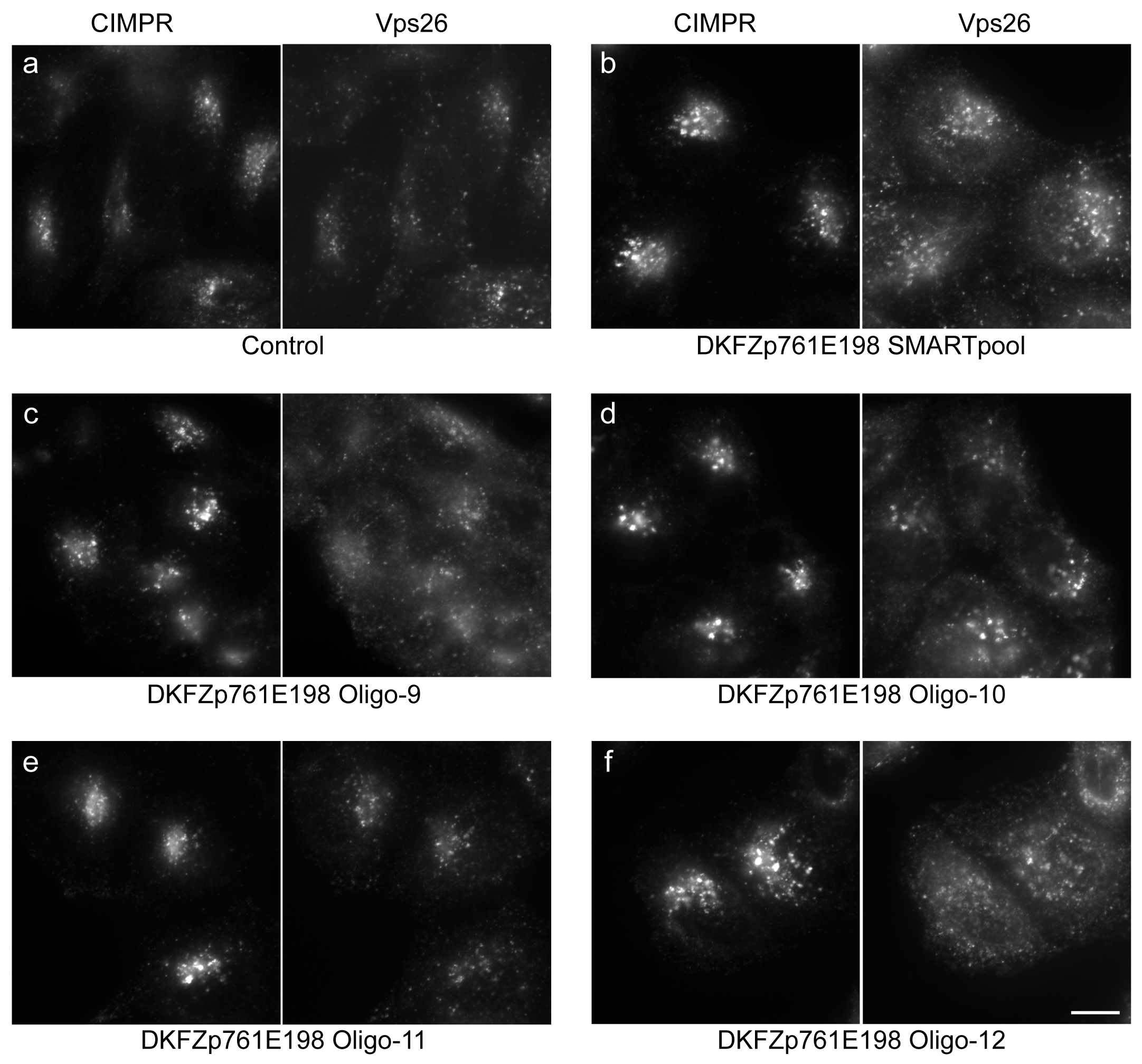

Supplement: Figure S3 — Phenotype of cells depleted of DKFZp761E198 using either a SMARTpool mixture of four siRNAs, or each siRNA individually. All four of the individual siRNAs, as well as the SMARTpool, change the localisation of the CIMPR in a similar manner to the C14orf108 siRNAs. Scale bar: 20 µm. (TIF) [file pbio.1001170.s003.tif]

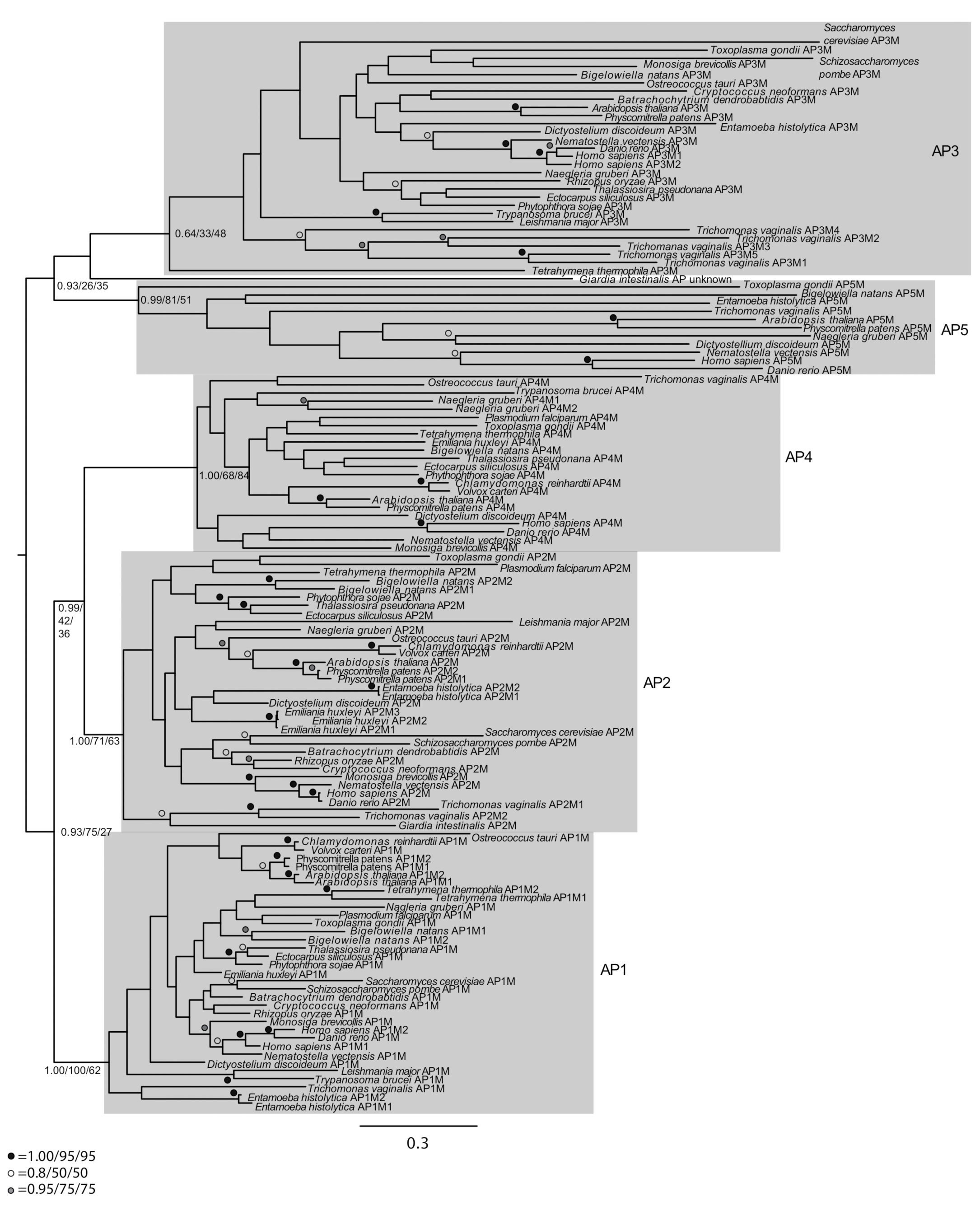

Supplement: Figure S4 — Phylogenetic analysis of all identified μ-adaptin homologues. In this and all subsequent figures the best Bayesian topology is shown, and values are given in the order of Bayesian posterior probabilities, PhyML derived maximum likelihood bootstrap values, and RAxML derived maximum-likelihood bootstrap values for the backbone nodes and those defining the adaptin protein families. Other values are replaced with symbols as inset. (TIF) [file pbio.1001170.s004.tif]

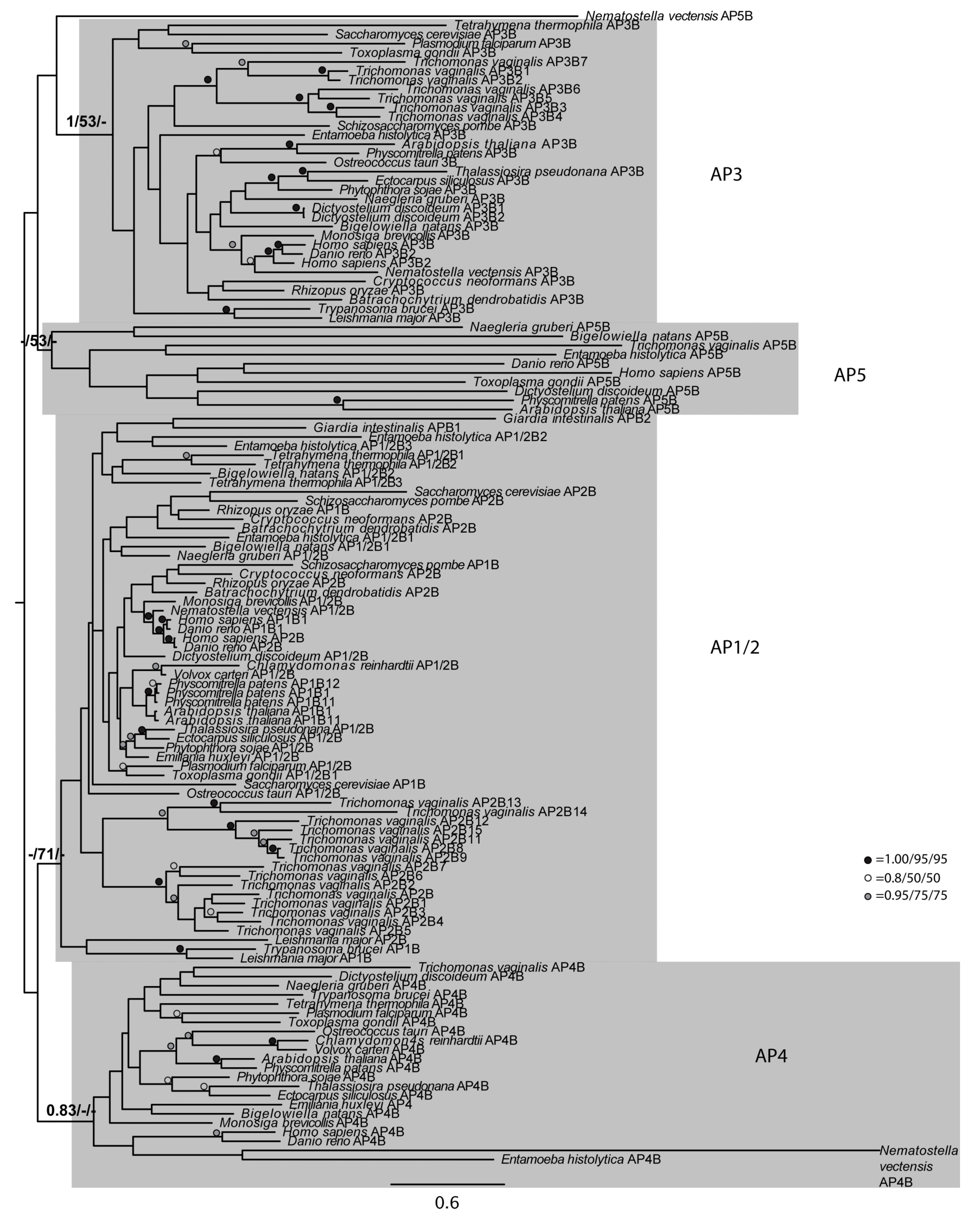

Supplement: Figure S5 — Phylogenetic analysis of all identified β-adaptin homologues. (TIF) [file pbio.1001170.s005.tif]

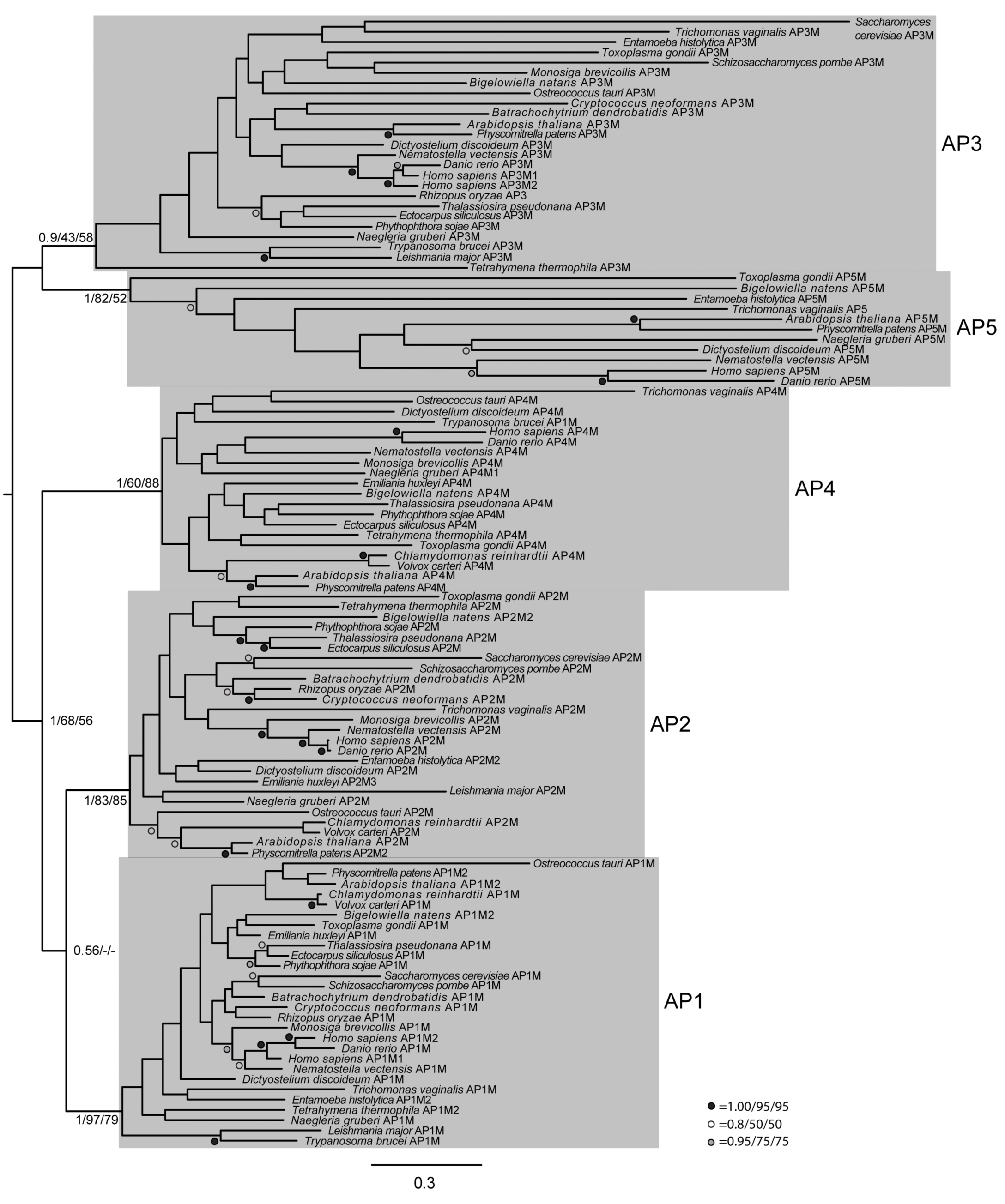

Supplement: Figure S6 — Phylogenetic analysis of μ-adaptin homologues with highly divergent and lineage-specific high identity duplicates removed. Note the robust support for the clade of μ5 orthologues. (TIF) [file pbio.1001170.s006.tif]

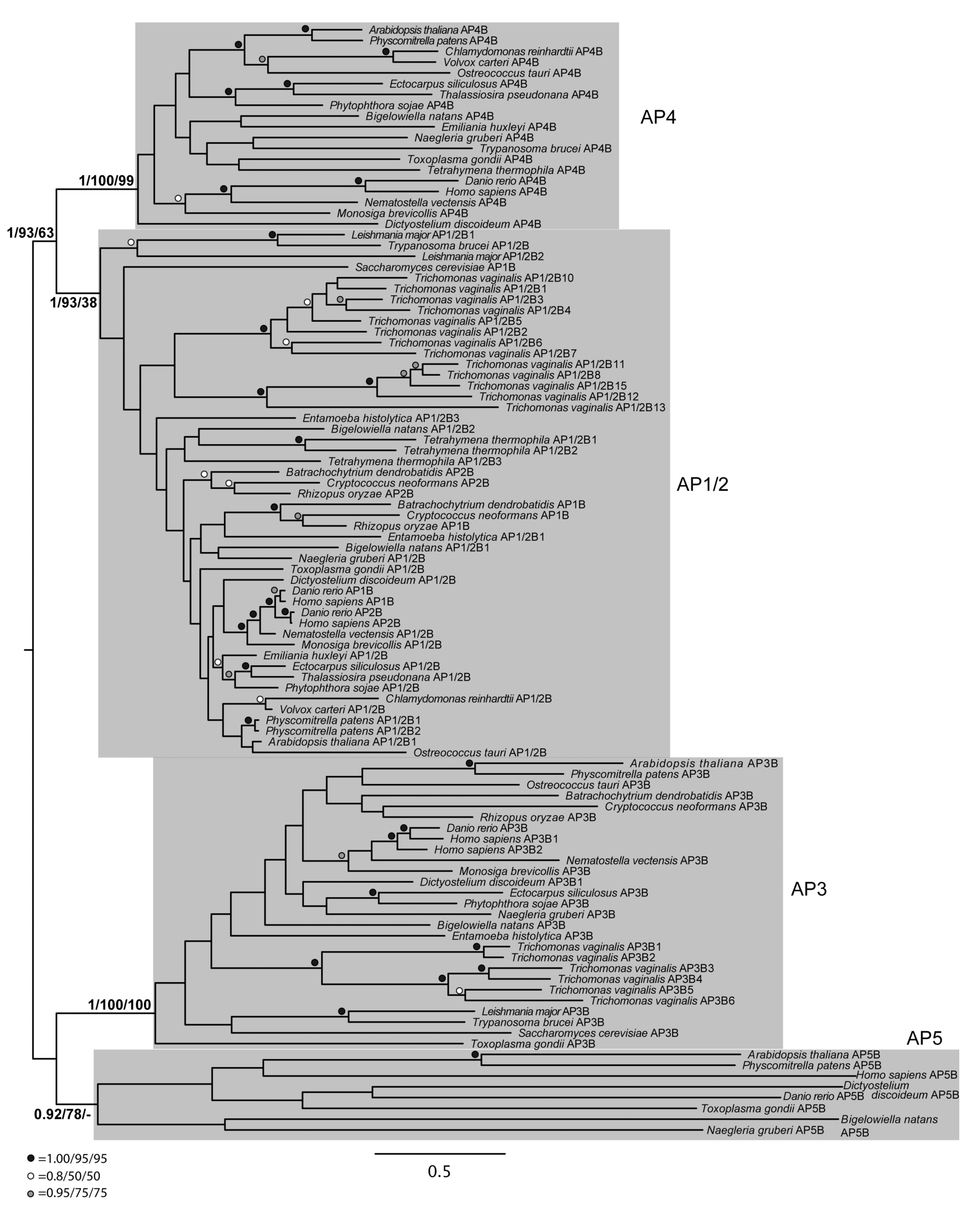

Supplement: Figure S7 — Phylogenetic analysis of β-adaptin homologues with highly divergent and lineage-specific high identity duplicates removed and including only putative homologues of β5 that were clearly identified using the PSI-BLAST criteria. (TIF) [file pbio.1001170.s007.tif]

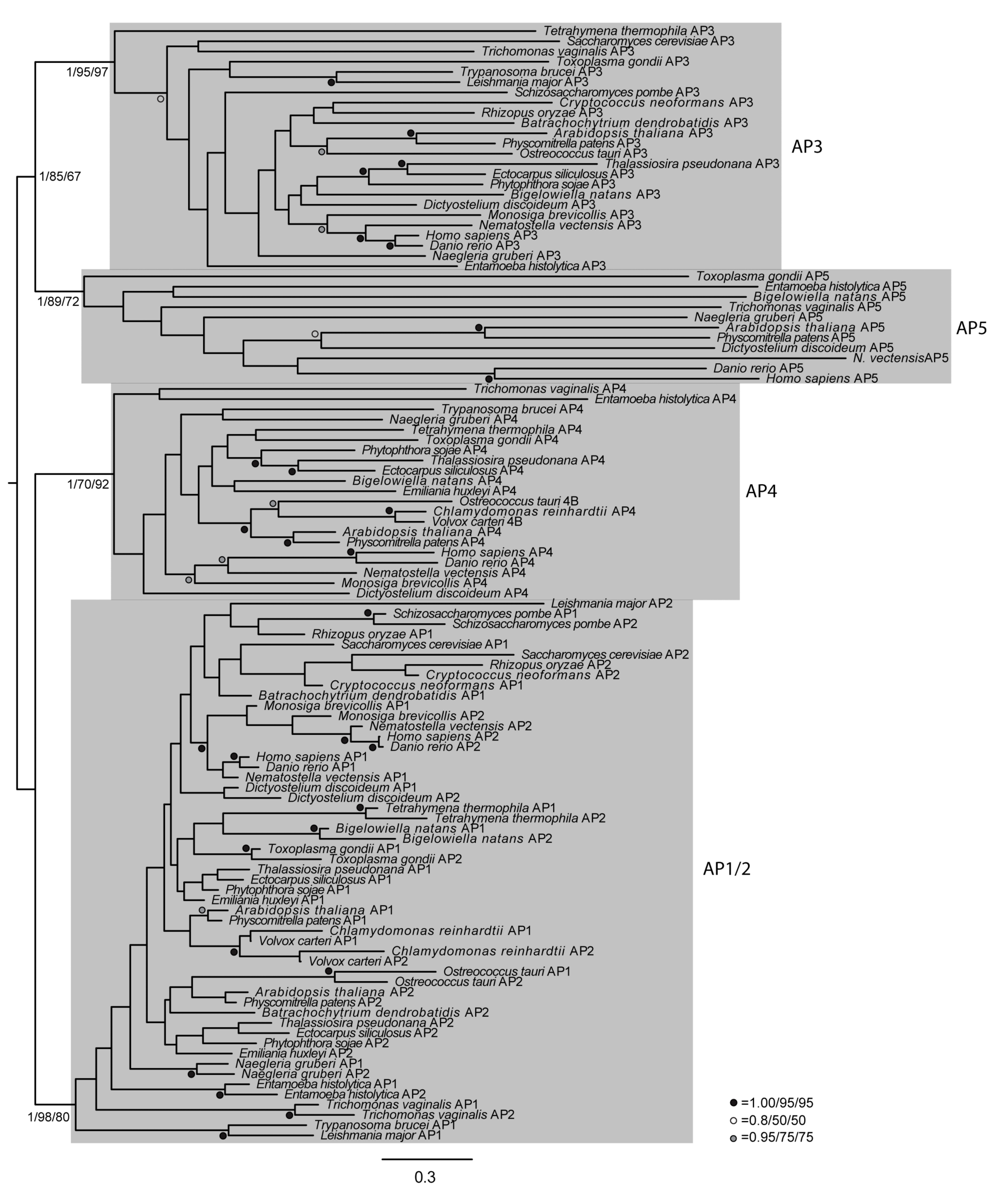

Supplement: Figure S8 — Phylogenetic analysis of a concatenated dataset of μ- and β-adaptin homologues. This encompasses all putative AP-5 homologues and robustly shows a clade of AP-5, thus solidifying their orthology. (TIF) [file pbio.1001170.s008.tif]

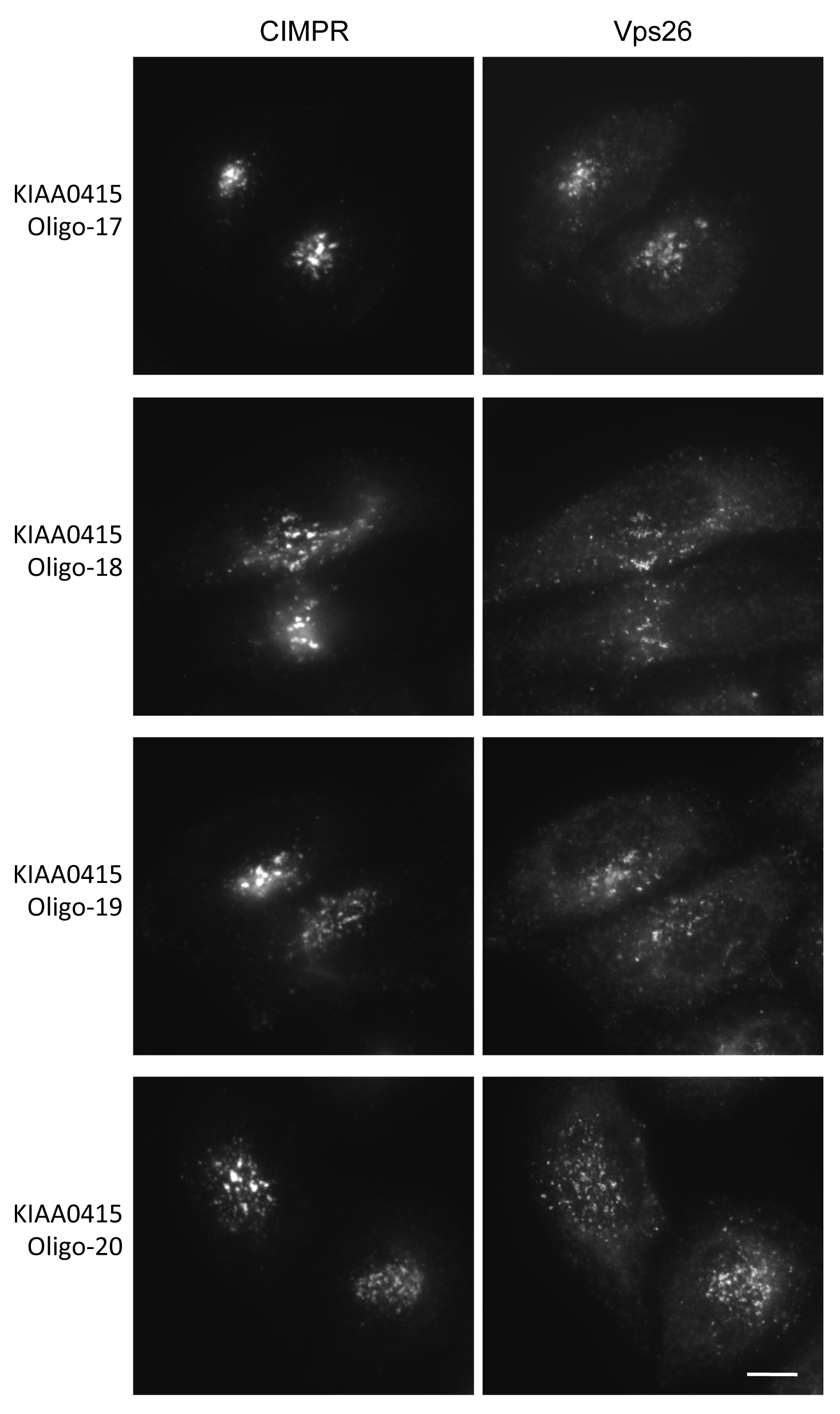

Supplement: Figure S9 — Phenotype of cells depleted of KIAA0415 using individual siRNAs instead of the SMARTpool. All four of the siRNAs change the localisation of the CIMPR. Scale bar: 20 µm. (TIF) [file pbio.1001170.s009.tif]

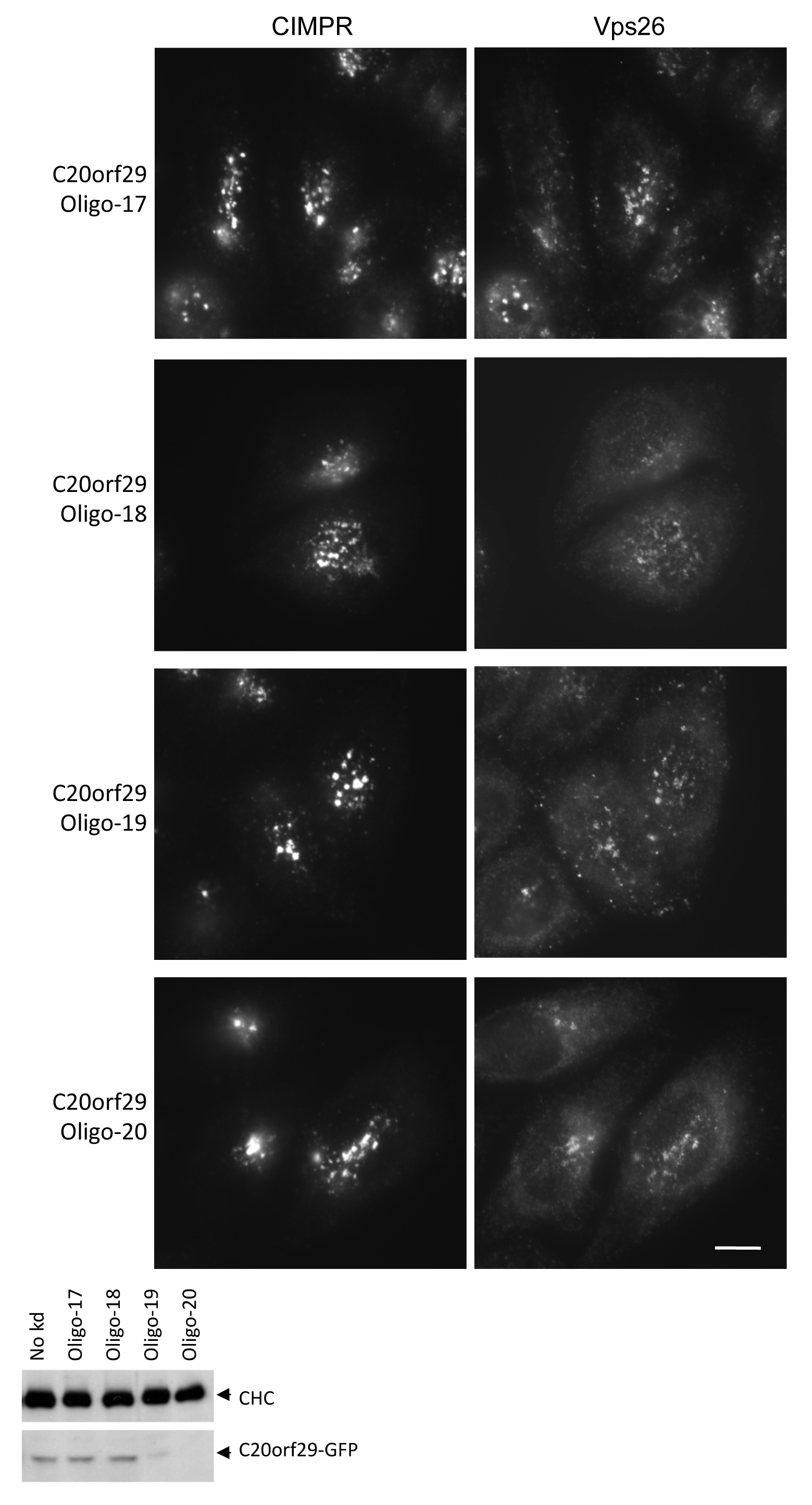

Supplement: Figure S10 — Phenotype of cells depleted of C20orf29 using individual siRNAs instead of the SMARTpool. (a) All four of the siRNAs change the localisation of the CIMPR. Scale bar: 20 µm. (b) The same four siRNAs were tested on cells transiently expressing GFP-tagged C20orf29, and the blot was probed with anti-GFP. The two siRNAs that target the coding sequence, Oligo-19 and Oligo-20, also deplete the GFP construct; however, Oligo-17 and Oligo-18, which target the 3′ UTR, do not deplete the construct because it has a different 3′ UTR. (TIF) [file pbio.1001170.s010.tif]

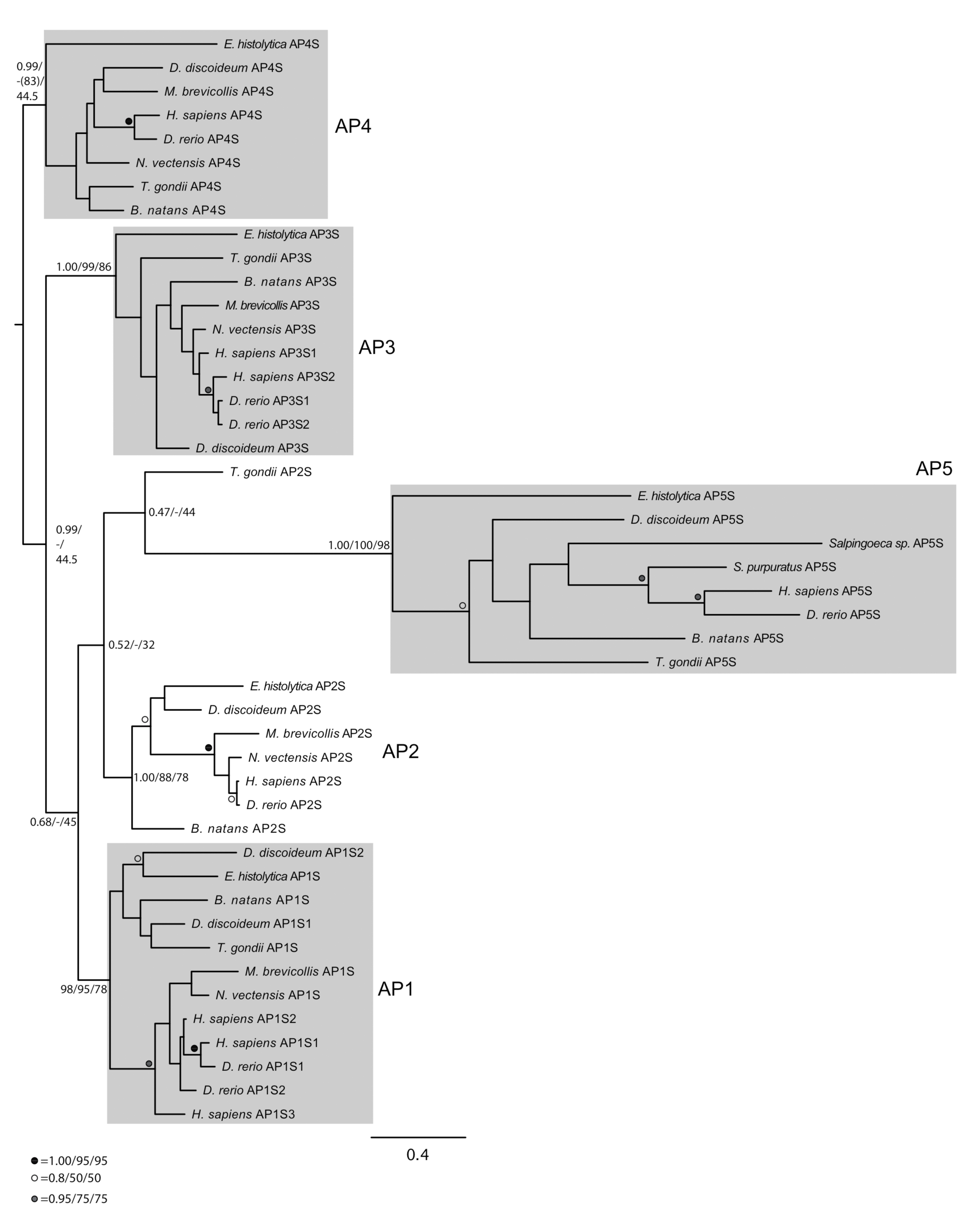

Supplement: Figure S11 — Phylogenetic analysis σ-adaptin homologues. Note the robust grouping of the putative σ5 orthologues including E. histolytica AP5S1 (XP_001914013.1), which retrieved the D. discoideum σ5 homologue as the most significant hit but with an e-value above the cut-off (0.082), thus validating the E. histolytica orthology as a σ5. (TIF) [file pbio.1001170.s011.tif]
